# Single-Photon DNA Photocleavage up to 905 nm by a Benzylated 4-Quinolinium Carbocyanine Dye

**DOI:** 10.1021/acsomega.4c07083

**Published:** 2025-02-11

**Authors:** Effibe O. Ahoulou, Aikohi Ugboya, Victor Ogbonna, Kanchan Basnet, Maged Henary, Kathryn B. Grant

**Affiliations:** 1 Department of Chemistry, Georgia State University, Atlanta, Georgia 30303, United States; 2 Center for Diagnostics and Therapeutics, Department of Chemistry, Georgia State University, Atlanta, Georgia 30303, United States

## Abstract

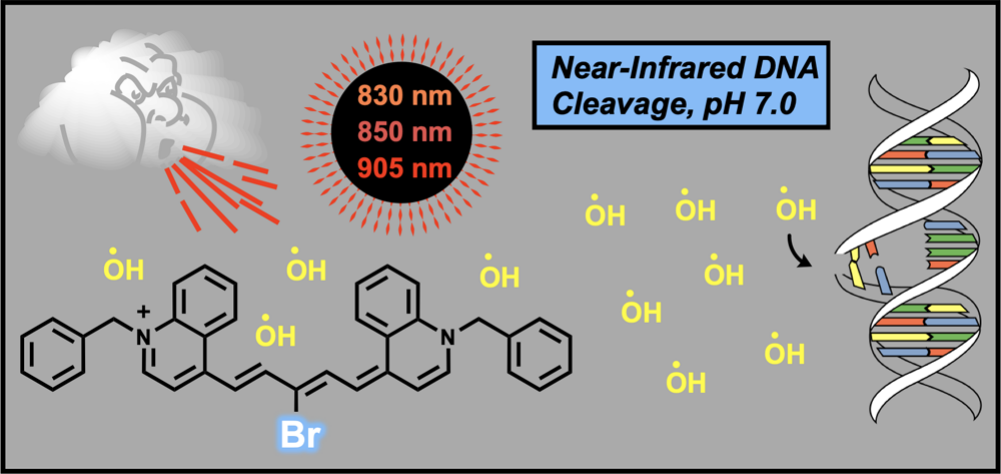

This paper describes the DNA interactions of near-infrared (NIR) benzylated 4-quinolinium dicarbocyanine dyes containing a pentamethine bridge *meso*-substituted either with a bromine (**4**) or hydrogen (**5**) atom. In pH 7.0 buffered aqueous solutions, the 4-quinolinium dyes absorb light that extends into the near-infrared range up to ∼950 nm. The unique direct strand breakage of pUC19 DNA that is sensitized by irradiating either dicarbocyanine with an 850 nm LED laser constitutes the first published example of DNA photocleavage upon single-photon chromophore excitation at a wavelength greater than 830 nm. Brominated dye **4**, which is more stable than and achieves DNA strand scission in higher yield than its hydrogen-bearing counterpart **5**, cleaves plasmid DNA under 830 and 905 nm laser illumination. The addition of increasing amounts of DNA to aqueous pH 7.0 solutions converted an aggregated form of dye **4** to a monomer with bathochromic absorption that overlaps all three laser emission wavelengths. No induced circular dichroism and fluorescence signals were detected when DNA was present, pointing to possible external binding of the dye to the DNA. Experiments employing radical-specific fluorescent probes and chemical additives showed that brominated dye **4** likely breaks DNA strands by photosensitizing hydroxyl radical production. Micromolar concentrations of the dye were relatively nontoxic to cultured *Escherichia coli* cells in the dark but dramatically reduced survival of the cells under 830 nm illumination. As NIR light wavelengths deeply penetrate biological tissues, we envisage the future use of carbocyanine dyes as a sensitizing agent in phototherapeutic applications.

## Introduction

Photodynamic therapy (PDT) is an emerging clinical tool that effectively treats medical conditions ranging from bacterial infections, early-stage inoperable cancers, and wet age-related macular degeneration to skin diseases such as psoriasis.^1−5^ The technique requires the intravenous, oral, or topical administration of a nontoxic chromophore called a photosensitizer (PS). Light is then used to activate the PS in targeted tissues, resulting in the highly localized production of short-lived, cytotoxic reactive oxygen species (ROS). This spatial and temporal control enables PDT to minimize side effects while maximizing treatment efficacy, by accurately targeting diseased tissues without compromising surrounding healthy cells. The therapy can be performed multiple times, before or after radiotherapy, chemotherapy, and surgery or in combination to enhance positive clinical outcomes.

In PDT, cytotoxic reactive oxygen species are generated by two different pathways.^1,2,4−6^ Upon excitation with an appropriate light source, the photosensitizer moves from its ground state to its triplet excited state (^3^PS*) through intersystem crossing from an intermediate excited singlet state (^1^PS*). The triplet states of many photosensitizers undergo type II energy transfer to ground state triplet oxygen (^3^O_2_) to generate the ROS singlet oxygen (^1^O_2_). Alternatively, in a type I process, the PS triplet excited state either directly, or upon reduction to an anion radical (^3^PS^•–*^), transfers an electron to ground state oxygen to generate superoxide anion radicals (O_2_^•–^). Spontaneous or enzymatic dismutation of the O_2_^•–^ then forms hydrogen peroxide (H_2_O_2_). The hydrogen peroxide, in turn, produces hydroxyl radicals (^•^OH) by interacting with trace Fe^2+^ in an oxidative process known as the Fenton reaction.^7,8^ With respective diffusion distances of 0.8–6.0 nm^9^ and 50–100 nm^10^ and half-lives of 1 ns^11^ and 3 μs*,*^12^ the highly reactive hydroxyl radicals and type II singlet oxygen thus formed^9,10^ in the overall PDT mechanism aggressively oxidize nearby macromolecules, such as proteins, nucleic acids, and lipids.^2,5,13^ This damage to biological molecules then affects targeted tissue-cell death through programmed (apoptotic) and/or nonprogrammed (inflammatory/necrotic) pathways.^13^

Though interest in PDT has grown swiftly over the past years, limitations accompanying deep-light penetration are prevalent among the majority of clinically approved photosensitizers. PDT agents in widespread use require visible-light sources with wavelengths that are readily absorbed by tissues, outside of the desired ∼700 to ∼900 nm optical window for efficient biological light transmission.^1,14,15^ As an example, the FDA-approved drug porfimer sodium (Photofrin) is activated at ∼630 nm^3^ and exhibits a low absorption coefficient of 1170 M^–1^ cm^–1^ at this wavelength, further limiting its effectiveness.^1,16^ Other routinely used photosensitizing agents include the porphyrin precursors δ-aminolevulinic acid (δ-ALA, Levulan; λ_ex_ = 405 or 635 nm) and methyl aminolevulinate (MAL, Metvixia; λ_ex_ = 630 nm) and the chlorins verteporfin (Visudyne; λ_ex_ = 689 nm), temoporfin (Foscan; λ_ex_ = 652 nm), and talaporfin (Talaporfin and Laserphyrin, λ_ex_ = 664 nm).^2−5^ With the relatively shallow penetration of red light, there is now great attention being focused on the development of new near-infrared (NIR) PDT agents that can be more efficiently activated in biological tissues.^13,17^

Cyanine dyes are frequently associated with low cellular dark toxicities and outstanding optical properties that include high molar absorption coefficients and strong florescence. As a result, indocyanine green (λ_ex_ = 800 nm; λ_em_ = 810 nm) and other cyanines are becoming important as clinical tools in a variety of medical imaging applications.^18−20^ The typical framework of a carbocyanine consists of two nitrogen-containing heterocyclic aromatic rings that share a positive charge through a connected, conjugated polymethine bridge. Fortuitously, cyanine absorption can be redshifted into the NIR range through extension of conjugation via the simple addition of more double bonds to the dye’s polymethine unit.^18^

Aromaticity and the delocalized positive charge of cyanines enable many of these dyes to interact with DNA as monomers and/or as aggregates.^21−25^ Several studies have reported using symmetrical 4-quinolinium,^15^ 2-quinolinium,^26^ and benz[*e*]indolium^27^ pentamethine carbocyanines, in addition to asymmetrical oxazole yellow (OY) and thiazole orange (TO)-based cyanine dyes^28−31^ to photosensitize the production of DNA cleaving ROS in the visible or near-infrared range. Some DNA photocleaving agents also known as “photonucleases” have impressive PDT applications. Porfimer sodium, talaporfin, and verteporfin, for example, directly cleave genomic DNA in circulating and/or in tissue culture cells under red light.^32−38^ Damage to mitochondrial (mt) DNA induces apoptosis, the sought-after, noninflammatory type of cell death that many highly proliferative cancer cells evade, thus making mtDNA a particularly appealing target in PDT.^39,40^ Given the propensity of cyanine dyes to localize within the mitochondria of cells, the development of cyanines as ROS-generating phototherapeutic agents in the near-infrared wavelength range is now of considerable interest.^13,17^

Although the transition from red to infrared wavelengths deepens the penetration of light through biological tissues, redshifting the λ_max_ of a chromophore has its drawbacks. The energy of the excited triplet state of the dye is reduced,^41^ which in turn limits near-IR photosensitized ROS production.^42^ For example, a PS should possess a triplet state energy ≥ the excitation energy of ^1^O_2_,^43^ which has led researchers to estimate that it should not be possible to generate type II ^1^O_2_ at wavelengths greater than ∼810 to ∼850 nm.^6,44^ For efficient type 1 superoxide formation, the triplet state oxidation potential of a photosensitizer should be greater than the ground state triplet oxygen oxidation potential.^45^ However, lowering triplet state energy reduces excited state oxidation potentials, hindering type I electron transfer reactions as well.^46^ As a result, there are relatively few examples of DNA photonucleases that are activated by light sources in the near-infrared wavelength range.^2^

Until this report, the upper wavelength limit to trigger the formation of direct DNA strand breaks upon single-photon chromophore activation was 830 nm.^15^ In an influential study, Chakravarty and his team reported high-yield, hydroxyl radical-mediated photocleavage of plasmid DNA by using single-photon 785 nm illumination to activate an anthracenyl-bis(pyridyl)Fe(III) PS.^47^ Zheng and co-workers then described efficient, two-photon DNA cleavage at 800 nm upon irradiating a carbazole methylpyridinium chromophore (*λ*_max_ = 418 nm) with a femtosecond pulsed laser.^48^ The use of two-photon techniques is not currently practical in most clinical settings, however, due to the requirement of costly ∼10 GW/cm^2^ fs pulsed laser with units that are powerful enough to damage biological specimens.^49^ In our laboratory, we had employed single-photon 3.3 W/cm^2^, 808 and 830 nm LED lasers in combination with a chlorinated 4-quinolinium carbocyanine dye to achieve hydroxyl radical-mediated cleavage of plasmid DNA (pH 7.0).^15^

**Scheme 1:**
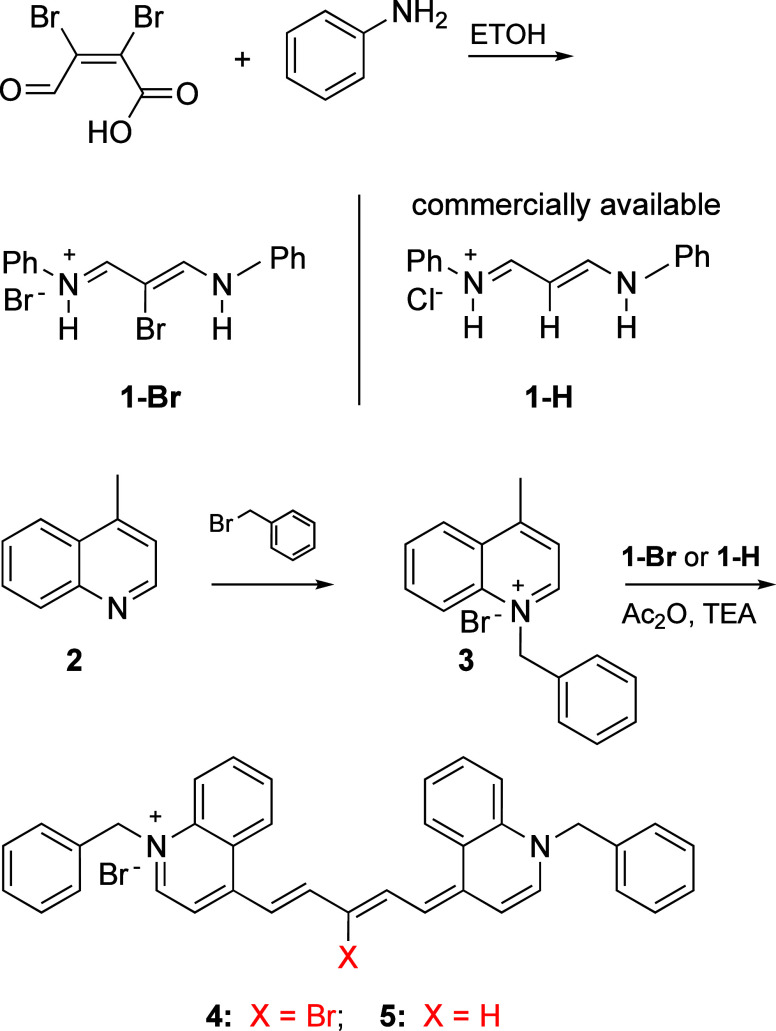
Synthesis of Carbocyanine Dyes **4** and **5**

To test the current wavelength limit for sensitizing single-photon oxidative DNA damage, here, we describe the synthesis of two symmetrical dicarbocyanine dyes in which redshifting benzylated 4-quinolinium rings are connected by a pentamethine bridge *meso*-substituted with either a bromine (**4**) or a hydrogen (**5**) atom (Scheme 1). Our analyses show that *meso*-brominated dye **4** cleaves DNA at 830, 850, and 905 nm and becomes highly phototoxic to cultured *Escherichia coli* (*E. coli*) cells under NIR illumination.

## Results and Discussion

### Dye Design and Synthesis

It was first necessary to acquire linkers for benzylated carbocyanine dyes **4** (X = Br) and **5** (X = H) (Scheme 1). While **1-H** (malonaldehyde bisphenylimine monohydrochloride) was purchased from a commercial source, the known compound **1-Br** was made by heating aniline with mucobromic acid in dry ethanol.^50,51^ Benzylated salt **3** was then synthesized by alkylating commercially available lepidine **2** with bromomethyl benzene at a 1:1 ratio. Salt **3** was heated at 70 °C for 1 h with linkers **1-Br** and **1-H** in acetic anhydride and triethylamine as a base. The formation of dyes **4** and **5** was monitored with a UV–vis spectrophotometer until peaks at ∼800–830 nm for dyes **4** and **5** were obtained and absorption at ∼450 nm disappeared. Upon cooling the reaction mixtures to room temperature, ethyl acetate was added, causing the dyes to precipitate. The resulting solids were filtered under vacuum and purified by solvation in a minimal amount of methanol (5 mL) and dilution with diethyl ether (10 mL). The crystals thus formed were filtered, dissolved in dichloromethane, and reprecipitated with hexanes. Each cyanine product was collected and dried under reduced pressure. Dyes **4** and **5** were both synthesized in good yields above 60% (Scheme 1).

The 4-quinolinium rings and the pentamethine bridges of dyes **4** and **5** were selected based on the 4-quinolinium photonuclease design that our laboratory developed to achieve single-photon DNA cleavage with light excitation at 808 and 830 nm.^15^ Toward the goal of photosensitizing the production of DNA-damaging ROS at longer wavelengths, the 4-quinolinium moieties of dyes **4** and **5** were benzylated. This was intended not only to redshift absorption but also to promote stabilizing van der Waals interactions between the dyes and the walls of the B-form DNA minor groove.^52^ One limitation of cyanine dyes, in particular those with long polymethine chains, is their propensity to slowly lose absorption in aqueous solutions due to spontaneous autoxidation (no *h*ν).^53^ To circumvent this complication, the electron-withdrawing *meso*-bromine atom in the pentamethine bridge of dye **4** was used to replace the hydrogen atom of dye **5**. The positioning of this halogen at the polymethine *meso*-carbon was intended not only to render dye **4** a poorer reducing agent but also to introduce a “heavy atom effect” in which ROS production would be optimized by increasing intersystem crossing rates to the dye’s excited triplet state.^54^

### Cyanine Stability and DNA Interactions

**Figure 1:**
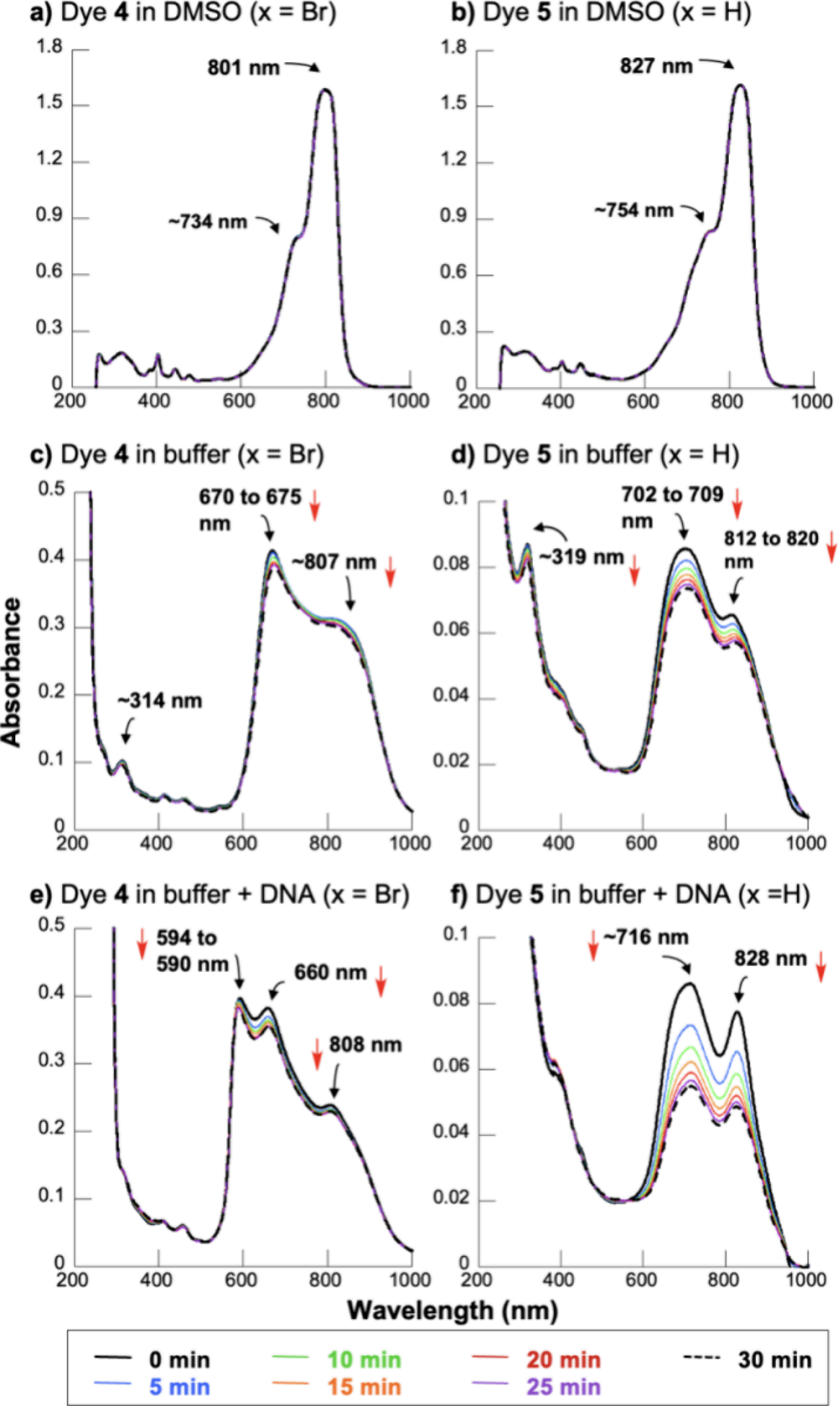
UV–visible spectra recorded over 30 min for 10 μM 4-quinolinium pentamethine carbocyanine dyes **4** and **5** in: (a,b) neat DMSO, (c,d) 10 mM sodium phosphate buffer pH 7.0, and (e,f) 10 mM sodium phosphate buffer pH 7.0 and 150 μM bp CT DNA (22 °C).

UV–visible spectra recorded as a function of time suggested that dyes **4** and **5** are stable in DMSO over 30 min, presenting sharp absorption bands with λ_max_ values of 801 nm ± 1 nm (**4**, X = Br) and 827 nm ± 2 nm (**5**, X = H) (Figure 1a,b). The observed wavelength reduction caused by the substitution of hydrogen for bromine was expected, as the introduction of electron-withdrawing groups to aromatic π systems imparts an inductive effect that reduces stabilizing electron delocalization.^55^ In addition to the main peaks at 801 nm (ε = 153,860 M^–1^ cm^–1^; Figure S1) and 827 nm (*ε* = 155,680 M^–1^ cm^–1^; Figure S1), dyes **4** and **5** in DMSO possess respective high energy shoulders at ∼734 and ∼754 nm. Consistent with the spectra shown in Figure 1a,b, the solvent DMSO is widely acknowledged to minimize cyanine aggregation in favor of the formation of dye monomers^15,23,26,56^ that are characterized by a sharp principal absorption band presented with a vibronic shoulder of higher energy.^57,58^ In addition to vibrational–electronic transitions, hypochromic features in cyanine spectra can arise from dye aggregation.^24^ To better define cyanine absorption, we therefore compared the DMSO spectra of **4** and **5** over dye concentrations ranging from 1.0 up to 10.0 μM (Figure S1 in the Supporting Information). For each spectrum, we then plotted the ratios obtained by dividing the absorption at the principal *λ*_max_ by the maximum absorption of its corresponding high energy shoulder (Figure S2). If dye aggregation had occurred, then an increase in cyanine concentration would be expected to cause a commensurate, steady decrease in the absorption ratios. As shown in Figure S2, the observed ratio changes from 1.0 up to 5.0 μM of **4** and **5** were relatively independent of the amount of the dye but decreased markedly at the highest dye concentration tested (10 μM). These results point toward the possibility of high energy cyanine dye vibronic transitions at low dye concentrations with additional hypsochromic absorption features arising from dye aggregation at concentrations approaching 10 μM. Notwithstanding, the sharp principal absorption bands that dominate the DMSO spectra of **4** and **5** suggest that the dyes are primarily in their monomeric forms in this solvent (Figure 1a,b and Figure S1).

Dyes **4** and **5** were synthesized in milligram quantities and then stored as concentrated 6–7 mM DMSO stock solutions at −20 °C (no hν). Upon transfer of diluted aliquots of the concentrated DMSO stocks to a neutral aqueous buffer, the peak absorption wavelengths of both dyes became blueshifted, pointing to a marked increase in dye aggregation (Figure 1c,d).^22−24^ Extensive autoxidation of dye **5** (X = H) in the aqueous buffer was indicated by its accelerated absorption loss over 30 min relative to dye **4**. In addition, dye **5** displayed extremely low bathochromic absorption at 0 min vs dye **4** accompanied by a relatively strong high energy absorption band (at ∼319 nm), suggesting that dye **5** may have decomposed upon aqueous sample preparation and/or during prolonged storage at −20 °C. Although both dyes were shown to be stable in DMSO over 30 min (Figure 1a,b), in contrast to dye **4**, we noted that the stored, concentrated DMSO stock solution of **5** tended to slowly lose absorption over time (−20 °C, no hν). Thus, the replacement of the *meso-*hydrogen for electron-withdrawing Br substantially stabilizes dye **4** (X = Br), making it more resistant to oxidation than its hydrogen-bearing counterpart (**5**).

When calf thymus (CT) DNA was added to the aqueous pH 7.0 buffered solutions, the positions and intensities of the main spectral bands of dyes **4** and **5** were altered, which is a strong indication that both dyes are independently binding to DNA (Figure 1e,f). Generally, interactions between cyanine dyes and DNA lead to dye–DNA complex formation that offers the dye greater stability toward autoxidation.^15,26^ However, we continued to observe absorption loss over time even when DNA was present, particularly in the case of dye **5**. This could be due to slow equilibration of dye/DNA interactions and/or to continued dye autoxidation.

### Dye-Photosensitized DNA Cleavage

We next employed a nondenaturing agarose gel electrophoresis nicking assay^59^ to test for the sensitized production of DNA-damaging ROS. In this technique, direct strand breaks in DNA are resolved due to a change in the conformation of plasmid DNA from a compact, supercoiled state to a slowly migrating, nicked form. The nondenaturing nicking assay does not detect alkaline labile lesions formed upon DNA oxidation and stable covalent DNA adducts. While hydroxyl radicals readily cleave DNA strands by abstracting hydrogen atoms from 2-deoxyribose,^60^ singlet oxygen forms mainly alkaline labile 8-hydroxy-2’-deoxyguanosine lesions,^61^ accompanied by minor amounts of direct strand breakage.^62,63^ There is very little if any published evidence to indicate that halogen radicals, superoxide anions radicals (O_2_^•–^), and hydrogen peroxide (H_2_O_2_) are sufficiently reactive to directly cleave DNA in aqueous solutions.^64−66^

**Figure 2:**
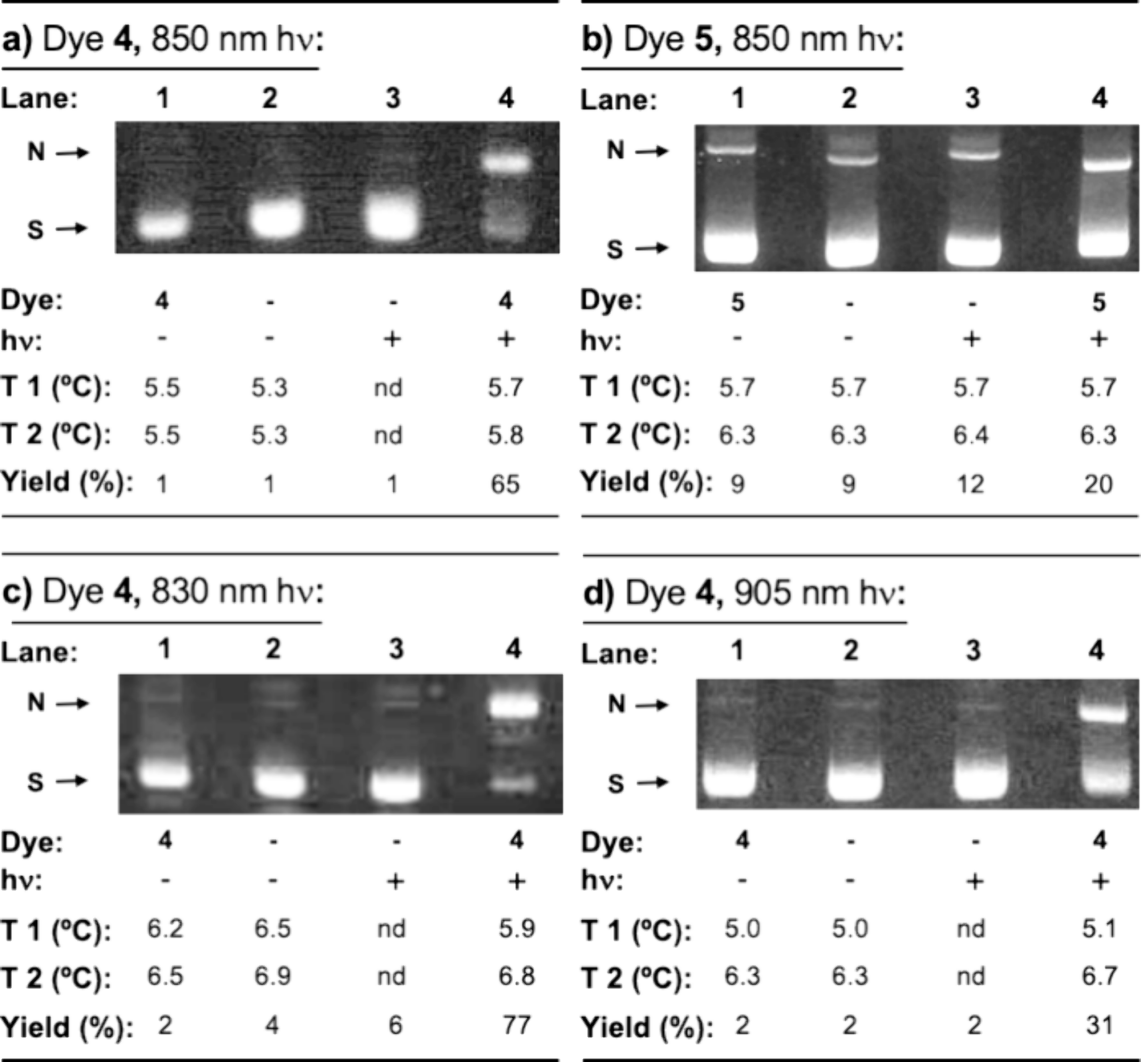
Representative ethidium bromide-stained 1.3% agarose gels showing photocleavage of pUC19 plasmid DNA. Reactions containing 10 mM sodium phosphate buffer pH 7.0 and 38 μM bp of pUC19 plasmid DNA in the presence and absence of 20 μM dye **4** or dye **5** were kept in the dark or irradiated for 30 min with an LED laser: (a,b) 850 nm, 0.9 W/cm^2^; (c) 830 nm, 3.3 W/cm^2^; or (d) 905 nm, 0.5 W/cm^2^. A metal block holding the samples was immersed in an ice bath during the reactions. Sample temperatures before (T1) and after (T2) the 30 min interval were recorded with a noncontact infrared thermometer. Following gel electrophoresis, cleavage yields were calculated by lane as described in Materials and Methods. Abbreviations: nd, not determined; N, nicked; S, supercoiled.

Our first nicking experiment was conducted with a single-photon NIR 850 nm, 0.9 W/cm^2^ LED laser overlapping dye absorption (Figure 1e,f). DNA photocleavage reactions contained plasmid DNA and 20 μM dyes **4** or **5** in sodium phosphate buffer pH 7.0. To minimize the effects of any heat generated by the LED light source, all samples were placed in a metal block immersed in an ice bath. The reactions in the chilled block were then covered with aluminum foil or irradiated for 30 min. Surface temperatures were measured with a thermometer before and after the 30 min interval. Samples were then electrophorized on nondenatured agarose gels. The photographic images of the gels in Figure 2a,b show that a small amount (∼20%) of supercoiled plasmid DNA was converted to nicked DNA upon irradiation of dye **5** (X = H), while dye **4** (X = Br) photocleaved the DNA in good yield (∼65%). As expected, minimal strand breakage was observed in all reactions protected from light. Relative to these dark controls, the temperatures of the irradiated samples did not substantially increase. This result rules against thermal processes as a major contributing phenomenon and instead suggests that the DNA strand breakage caused by dyes **4** (X = Br) and **5** (X = H) is photochemically driven. The inability of dye **5** to induce high levels of DNA cleavage at 850 nm is consistent with its low amount of UV–visible absorption and apparent rapid autoxidation rate in an aqueous buffer (Figure 1d,f). Due to this stability issue, we discontinued work with dye **5** and employed dye **4** (X = Br) in all remaining studies.

The photocleavage experiment just described was then repeated using single-photon 830 nm, 3.3 W/cm^2^ and 905 nm, 0.5 W/cm^2^ LED lasers to activate brominated dye **4**. The resulting gels in Figure 2c,d reveal dye-sensitized direct strand scission of DNA at both wavelengths. When all three light sources are compared, photocleavage yields are found to be consistent with both LED laser power and the relative absorption of light by the DNA/complex (830 nm > 850 nm > 905 nm; Figures 1e and 2). To the best of our knowledge, these data represent the first reported examples of direct DNA strand breakage upon single-photon chromophore excitation at wavelengths greater than 830 nm.

**Figure 3:**
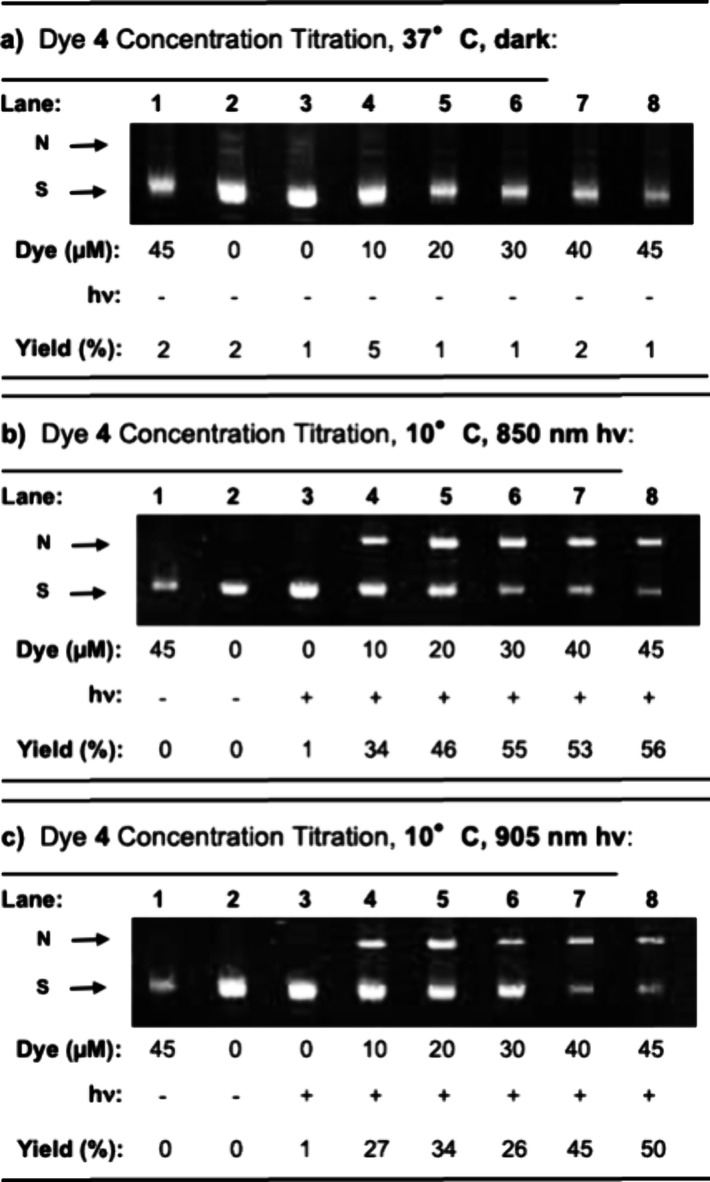
Representative ethidium bromide-stained agarose gels depicting photocleavage of 38 μM bp of pUC19 plasmid DNA in the presence of dye **4** concentrations ranging from 0 up to 45 μM (10 mM sodium phosphate buffer pH 7.0). The reactions were either (a) heated for 30 min at 37 °C in the dark or cooled for 30 min in an ∼10 °C metal block either in the dark (-) or while being irradiated with (b) 850 nm, 0.9 W/cm^2^ or (c) 905 nm, 0.5 W/cm^2^ LED laser (+). Following gel electrophoresis, cleavage yields were calculated by lane as described in Materials and Methods. Abbreviations: N, nicked; S, supercoiled.

DNA cleavage at dye **4** concentrations ranging from 10 up to 45 μM was tested next. Individual reactions were either heated at 37 °C in the dark or cooled to ∼10 °C and then irradiated at 850 or 905 nm (30 min, pH 7.0; Figure 3). The results of these experiments show that **4** is unable to cleave DNA upon prolonged heating of dark reactions at a physiological temperature of 37 °C (Figure 3a). As expected, on irradiation of reactions, direct DNA strand breakage was observed at levels that generally were raised as a function of increasing dye **4** concentration (Figure 3b,c). With the 850 nm LED laser, a cleavage plateau was reached at ∼30 μM of the dye, whereas at 905 nm, strand breakage incrementally increased up to ∼45 μM (apart from an outlying data point). However, it is important to note that the fluorescence of the ethidium bromide probe used to visualize the DNA appears to decline at dye concentrations ≥40 μM (Figure 3, lanes 7 and 8). This suggested that some cyanine dye might have remained DNA-bound during electrophoresis at these high concentrations, preventing ethidium bromide from intercalating into the DNA, thus decreasing its fluorescence and introducing error into the estimation of the corresponding reaction yields reported in Figure 3. As a result, we avoided utilizing dye concentrations ≥40 μM in future experiments involving ethidium bromide-stained gels.

**Figure 4:**
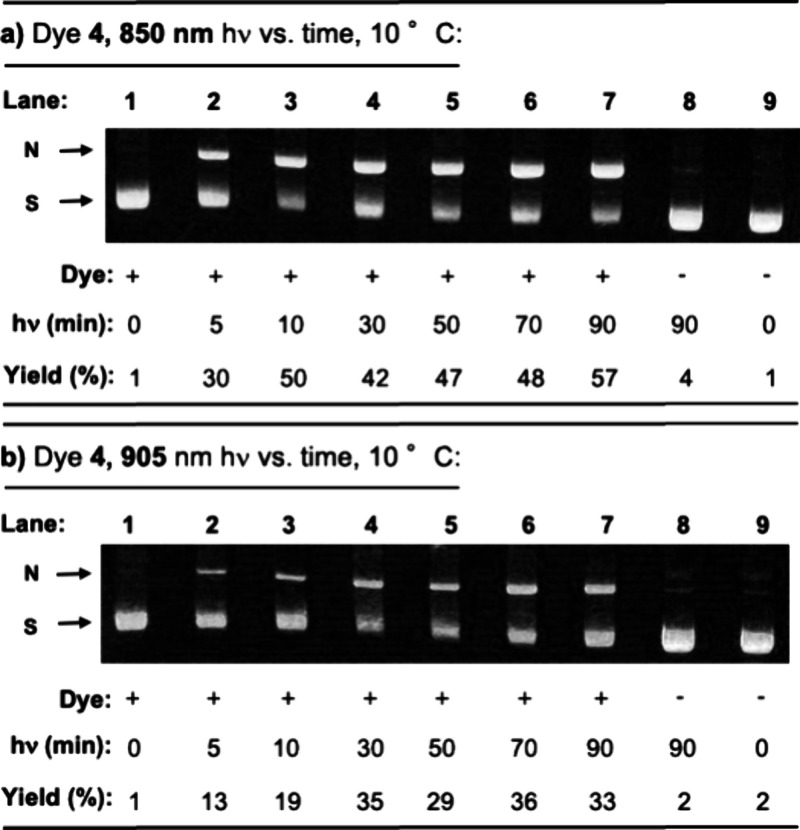
Representative ethidium bromide-stained agarose gels showing photocleavage of 38 μM bp of pUC19 plasmid DNA in reactions containing 10 mM sodium phosphate buffer pH 7.0 in the presence and absence of 20 μM dye **4**. In ∼10 °C metal blocks, the reactions were irradiated for intervals of time ranging from 0 up to 90 min with (a) 850 nm, 0.9 W/cm^2^ or (b) 905 nm, 0.5 W/cm^2^ LED laser. Following gel electrophoresis, cleavage yields were calculated by lane as described in Materials and Methods. Abbreviations: N, nicked; S, supercoiled.

We next employed 20 μM of dye **4** to photocleave plasmid DNA at irradiation times ranging from 0 to 90 min (10 °C, pH 7.0). The gel image in Figure 4b demonstrates that dye **4** requires ∼30 min to achieve steady levels of DNA damage under 905 nm, 0.5 W/cm^2^ illumination. In contrast, maximum cleavage yields are higher after only ∼10 min in the case of the more powerful 850 nm, 0.9 W/cm^2^ light source (Figure 4a). These results suggest that the same ROS that are generating direct strand breaks in DNA may also be reacting with dye **4**, causing it to lose activity as a function of time.

### DNA Binding Mode Analyses

Cyanine dye aggregation is frequently observed in aqueous solutions. Compared to the spectral properties of cyanine monomers, cofacial H-aggregates have absorption maxima that are blueshifted, while staggered cyanine J-aggregates have narrow, redshifted absorption bands.^24^ Depending on its structural characteristics, a carbocyanine can bind to DNA in its monomeric and/or H- and J-aggregated forms either externally and/or in the narrow B-form minor groove.^21−23,67^ While intercalative DNA binding is possible for cyanine monomers, it is highly unlikely to occur in the case of the H- and J-forms due to the self-stacking of the dyes’ intercalating aromatic rings within the aggregate structure. To better understand the relationship between cyanine dye **4** aggregation and DNA binding mode, additional spectroscopic measurements were recorded.

**Figure 5:**
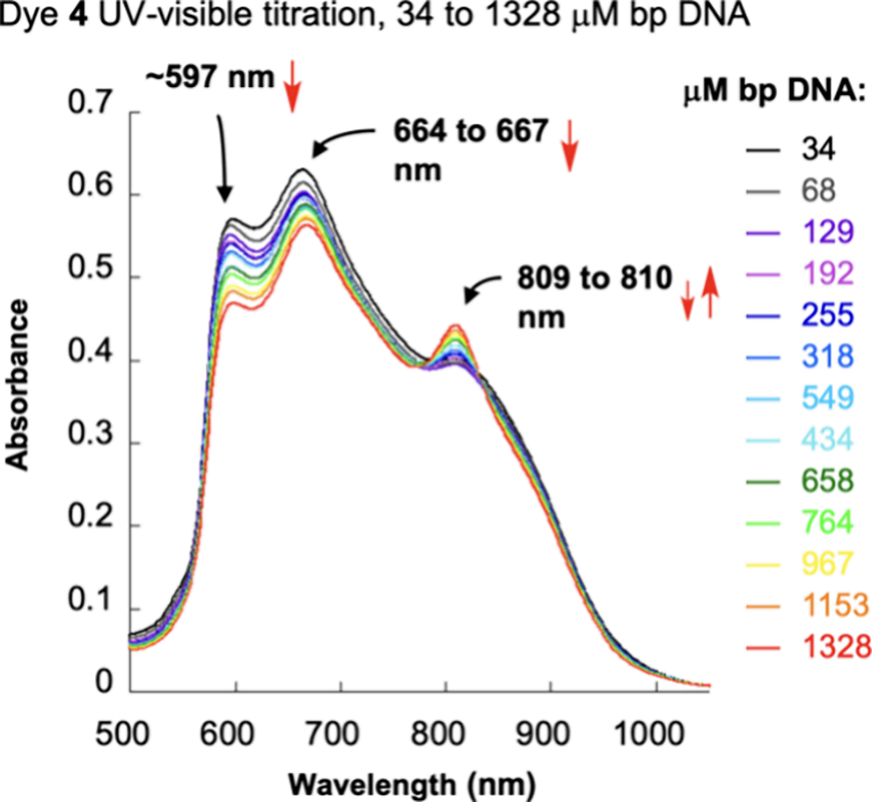
Representative UV–visible absorption spectra of solutions containing 20 μM dye **4** and 10 mM sodium phosphate buffer pH 7.0 in the presence of increasing CT DNA concentrations from 34 μM up to 1328 μM bp (22 °C).

In our first experiment, increasing concentrations of calf thymus (CT) DNA were incrementally transferred to the aqueous pH 7.0 buffered solution containing a fixed amount of dye **4**. The UV–visible spectra in Figure 5 reveal subtle changes in the dye’s aggregation patterns, which are small in magnitude compared to the behavior of other symmetrical 2- and 4-quinolinium cyanine dyes studied by our laboratory.^15,26^ As more CT DNA is added, the intensities of dye **4**’s absorption bands at ∼597 and ∼664–667 nm decrease, peaks that likely are composed of the superimposed spectra of free and DNA-bound dye aggregates. A number of research groups including our own have correlated DNA addition to the gradual conversion of cyanine aggregates to DNA-bound monomers.^15,21,22,25,26^ In this regard, as DNA is added, in Figure 5, we observe an initial small decrease followed by a more substantial increase in dye **4**’s ∼809–810 nm peak, which is similar in λ_max_ to the putative monomeric form of **4** in DMSO (801 nm; Figure 1a).

Interactions of an achiral cyanine with chiral DNA can generate an induced circular dichroism (ICD) signal that provides information about the DNA binding mode of the dye. For example, ICD signals are usually observed in the case of DNA groove binding when adjacent end-to-end groove-bound cyanine dimers and higher-order aggregates exhibit strong bisignate exciton coupling.^21−23^ Alternatively, groove-bound monomers and simple groove-bound dimers are associated with positive ICDs,^22,68^ while the ICD signals of cyanine intercalators can be positive^68^ or negative^25,67,69^ and are usually relatively weak. In contrast to minor groove and intercalative DNA binding modes, induced CD signals are frequently absent when achiral ligands interact with B-form DNA in an external fashion.^25,70−74^

**Figure 6:**
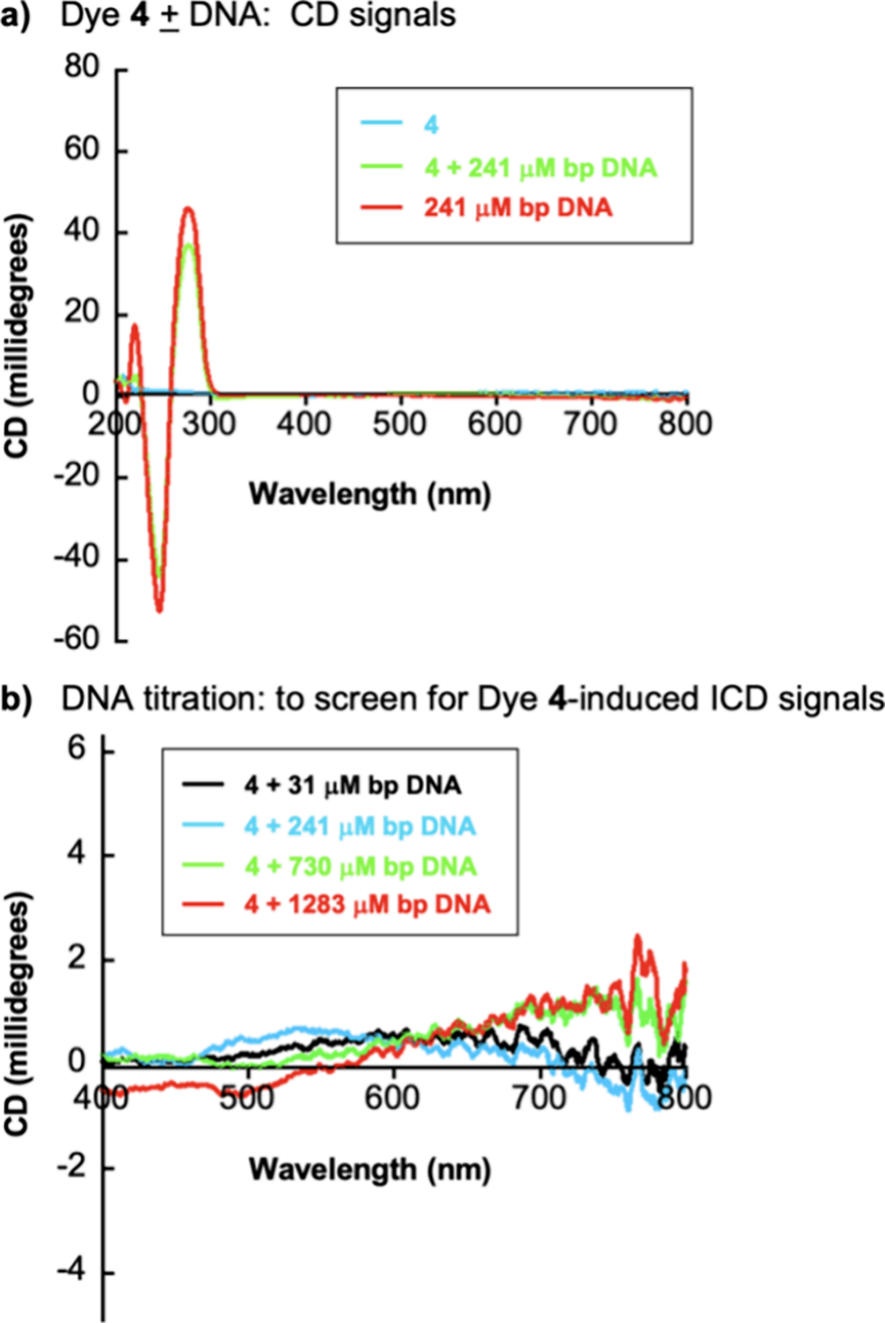
Circular dichroism (CD) spectra of solutions containing 10 mM sodium phosphate buffer pH 7.0, 20 μM of dye **4**, and/or (a) 241 μM bp of CT DNA; (b) CT DNA concentrations ranging from 31 μM up to 1283 μM bp (22 °C).

To further evaluate DNA/carbocyanine dye interactions, CD spectra of dye **4** were recorded from 200 to 800 nm in an aqueous pH 7.0 buffer in the presence of increasing concentrations (0–1283 μM bp) of CT DNA (Figure 6). As expected, achiral dye **4** has no CD signal, while the CT DNA molecules exhibit consecutive negative and positive CD bands between 200 and 300 nm due to the chirality arising from its right-handed B-form structure.^75^ Upon adding the CT DNA to dye **4**, even at concentrations as high as 1283 μM bp, no ICD signals corresponding to the absorption bands of the putative aggregated and monomeric DNA-bound forms of the dye are seen (Figure 6). Although CD signals could not be acquired above 800 nm due to detector sensitivity, typical peak broadening arising from vibrational band overlap would be expected to produce a bell-shaped spectrum extending beneath the 809–810 nm λ_max_ of the DNA-bound monomer into the range of our CD detector. Taken together, the CD results suggest that dye **4** likely engages in outside edge interactions with the DNA backbone (external binding). In the case of the monomeric form of dye **4**, intercalation cannot be completely ruled out at this point due to the very weak ICD signals often associated with cyanine intercalators.^25,67,69^

**Figure 7:**
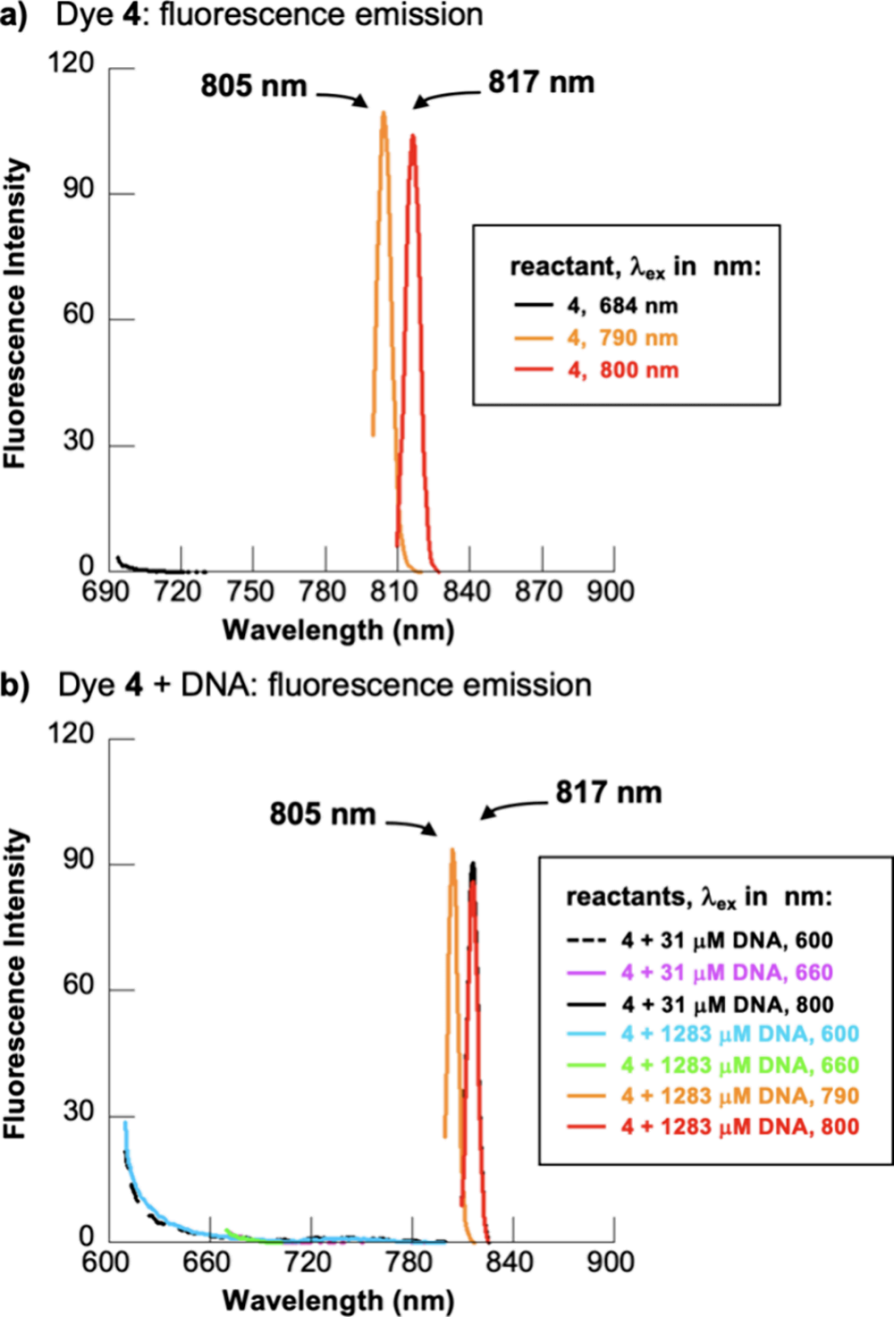
Fluorescence emission spectra acquired at excitation wavelengths (λ_ex_) of (a) 684, 790, and 800 nm for 10 μM dye **4** and (b) 600, 660, 790, and 800 nm for 10 μM dye **4** in the presence of 31 μM or 1283 μM bp of CT DNA (10 mM sodium phosphate buffer pH 7.0, 22 °C).

Cyanine dyes that intercalate often exhibit large increases in fluorescence upon interacting with DNA by restricting the conformational twisting of the cyanine, thereby preventing rapid nonradiative deactivation of its singlet excited state.^25,76,77^ With the intent of further evaluating the mode of interaction of the brominated cyanine dye, fluorescence spectra of 10 μM dye **4** were recorded in the presence of 0, 31, and 1283 μM bp of CT DNA concentrations (Figure 7). Various excitation wavelengths that overlap with the absorption of the aggregated and monomeric forms of free and bound dye **4** were evaluated (Figures 1 and 5). In all cases, fluorescence emission by dye **4** was not detected, irrespective of the presence of DNA. This being said, we have identified the narrow bands at 805 and 817 nm in Figure 7 as light scattering artifacts based on the following reasoning. In general, λ_em_ values are independent of excitation wavelength because the first excited singlet state (S_1_) lifetimes of most fluorophores are long enough to allow relaxation from higher S_1_ vibrational energy levels (e.g.*,* by internal conversion) to the lowest S_1_ state prior to emission.^78^ When we increased 790 nm excitation by 10 nm up to 800 nm for reaction samples containing dye **4** in the presence and absence of CT DNA, the putative emission peaks in Figure 7 were shifted from 805 to 817 nm, a hallmark of Stokes Raman light scattering.^78^ While aggregated cyanines cannot bind to DNA by intercalation, the absence of a fluorescence signal upon excitation of the 809 to 810 nm DNA-bound absorption peak in Figure 5 suggests that the monomeric form of dye **4** is not intercalated within DNA, a hypothesis that is consistent with our circular dichroism measurements showing that there is no ICD signal in this region (Figure 6).

**Figure 8:**
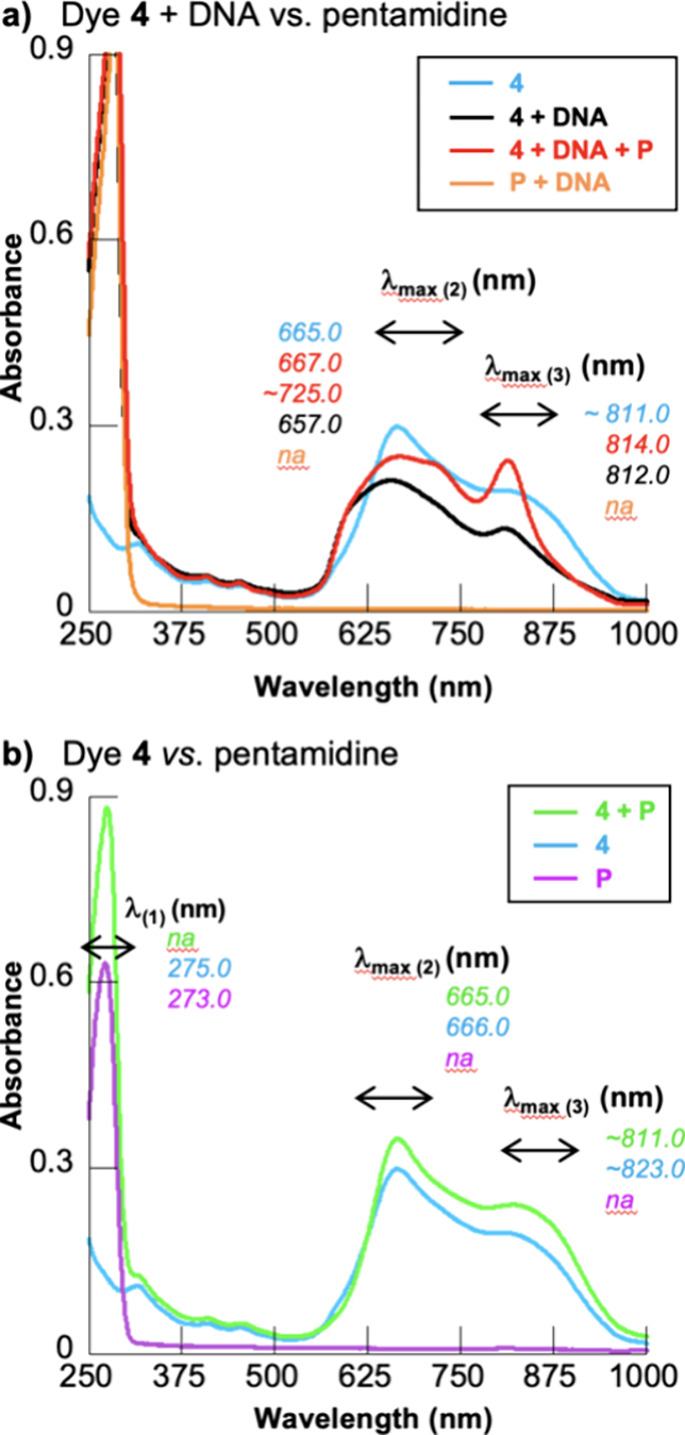
UV–visible absorption spectra of solutions containing 10 mM sodium phosphate buffer pH 7.0 with combinations of (a) 10 μM dye **4**, 50 μM pentamidine (P), and 150 μM bp of CT DNA; (b) 10 μM dye **4** and 50 μM pentamidine. Abbreviation: na, not applicable.

Pentamidine is a well-known antimicrobial agent used to treat a variety of infections including leishmaniasis, African trypanosomiasis, and *Pneumocystis carinii* pneumonia. Its structure, which consists of two 4-amidinophenyl groups connected by a flexible pentane-1,5-diol linker, promotes snug binding of this compound within the minor groove of AT-rich regions of B-form DNA.^79,80^ The reported equilibrium dissociation (*K*_D_) constant of pentamidine for duplex DNA containing the sequence AATT is 7.69 μM at pH 7.0. Footing printing experiments have shown that pentamidine binds to DNA sites containing five or more consecutive AT base pairs with higher affinity than sites consisting of four AT base pairs, without distinguishing between homopolymeric and alternating sequences.^81^ Unlike the many intercalators that significantly lengthen and unwind DNA,^82^ changes to DNA duplex structure caused by pentamidine are minimal,^79,80^ making it a good compound to test dye **4** for minor groove interactions. Toward this end, UV–visible spectra of solutions containing different combinations of dye **4**, pentamidine, and CT DNA were recorded (pH 7.0, 22 °C; Figure 8).

When DNA was present, our results show that pentamidine addition produced noticeable changes to the spectral features of the dye/DNA complex (Figure 8a, black line): the λ_max_ of the putative dye monomer was shifted from 812 to 814 nm and exhibited major hyperchromicity (Figure 8a, red line); a local maximum tentatively assigned as a dye aggregate appeared as a shoulder at ∼725 nm (Figure 8a, red line). When DNA was absent, pentamidine addition altered the UV–visible spectrum of dye **4**, but to a lesser extent (Figure 8b, green line). General hyperchromicity and some wavelength shifting were observed, pointing to the possibility of direct interactions between dye **4** and pentamidine. While the experimental results shown in Figure 8 suggest that the binding of pentamidine to the DNA minor groove alters how dye **4** interacts with DNA, there appears to be little of any release of the free dye from the DNA upon pentamidine addition (Figure 8a). Release of **4** would be expected to occur if the dye were to be bound to AT-rich sequences in the DNA minor groove in a similar fashion to pentamidine, distamycin, and other classical groove binding molecules that favor the steric factors afforded by narrow AT-rich B-form minor groove widths.^79,80^ When taken together with the data obtained from our CD and fluorescence experiments, there is little if any evidence that dye **4** engages in a significant degree of intercalative and/or classical minor groove binding interactions with DNA (Figures 6, 7, and 8).

### Analyses and Identification of Photosensitized ROS

In these experiments, possible mechanisms underlying dye **4**-photosensitized DNA cleavage were explored by using chemicals to modify levels of photosensitized direct DNA strand breakage, e.g*.,* the minor groove binder pentamidine; sodium benzoate, a scavenger that quenches hydroxyl radicals (^•^OH) at near diffusion-controlled rates;^83^ deuterium oxide, which increases the half-life of type II singlet oxygen 10-fold;^84^ and the metal chelator ethylenediaminetetraacetate (EDTA). Reactions consisting of pUC19 plasmid DNA and dye **4** in the presence and absence of one of the above-listed additives were irradiated at 850 nm for 30 min and then resolved on agarose gels. The extent of DNA photocleavage inhibition caused by each of the additives was then estimated by integrating gel photographs (Table 1 and Figures S3 and S4 in the Supporting Information).

**Table 1:** DNA Photocleavage Inhibition Assays^a^

**reagents added**	**target**	**cleavage inhibition (%)**
pentamidine	minor groove	22 ± 10
Na^+^ benzoate	^•^OH	53 ± 0.3
EDTA	Fe^2+^, Fe^3+^/Cu^1+^, and Cu^2+^	57 ± 12
D_2_O	^1^O_2_	7 ± 2

a To reactions containing 10 mM sodium phosphate buffer pH 7.0, 38 μM bp of pUC19 plasmid DNA, 30 μM of dye **4**, and a final concentration of one of the following reagents were added: sodium benzoate (100 mM), EDTA (100 mM), 79% D_2_O *(v*/*v),* or pentamidine (50 μM). The samples were kept in dark or were irradiated for 30 min alongside control reactions with no additive using an 850 nm, 0.9 W/cm^2^ laser (22 °C). Cleavage inhibition yields were averaged over 2–3 trials with error reported as standard deviation. Representative gels are in Supporting Information (Figures S3 and S4).

Upon irradiation of dye **4**, our results show that pentamidine reduces the formation of DNA direct strand breaks by ∼22% (Table 1 and Figure S3), a finding that is consistent with our hypothesis that this classical minor groove binder alters interactions between dye **4** and DNA (Figure 8). The hydroxyl radical scavenger sodium benzoate and the metal ion chelator EDTA respectively inhibit direct DNA breakage by ∼53 and ∼57%. When water was replaced with 79% D_2_O (v/v), rather than enhancing DNA photocleavage, the deuterated solvent reduced strand breakage by ∼7%. Similar results were reported by Williams and co-workers during their investigation of the DNA photonuclease activity of a copper(II) hexaazatriphenylene-based complex. In their study, DNA photocleavage inhibition by D_2_O was attributed to the possibility that hydroxyl radical production by the Cu(II) complex had been mitigated by a deuterium isotope effect.^74^ Taken together, the results of our chemical additive experiments suggest that, rather than singlet oxygen, hydroxyl radicals generated through metal-assisted Fenton chemistry are major contributors to dye **4**-sensitized DNA photocleavage (Table 1 and Figure S4).^7,60^ Adventitious levels of redox-active copper and iron ions are commonly present in aqueous laboratory solutions,^85−87^ including but not limited to deionized (DI) water.^88^ Due to their minute concentrations, these ions are exceedingly difficult to quantitate and eliminate. Common sources include metals that leach from equipment and plasticware^85,86,89^ and from the aerosols and fumes present in the ambient air of conventional laboratories.^85^ It is conceivable that metal binding sites in the cloned pUC19 DNA in our DNA photocleavage experiments had sequestered trace amounts of metal ions during the alkaline lysis step that we used to isolate and purify the plasmid from *E. coli*.^87^ Aside from *in vitro* laboratory reactions, redox-active copper and iron ions engage in Fenton chemistry to form harmful hydroxyl radicals in living systems. Conditions of oxidative stress triggered by ischemia and reperfusion injury,^90,91^ exposure to environmental toxins,^92^ and other insults form hydroxyl radicals that burst lysosomes^93^ and inflamed, necrotic cells, releasing weakly chelated pools of iron and copper ions that disseminate hydroxyl radical-mediated damage to surrounding healthy tissues.^91^

**Figure 9:**
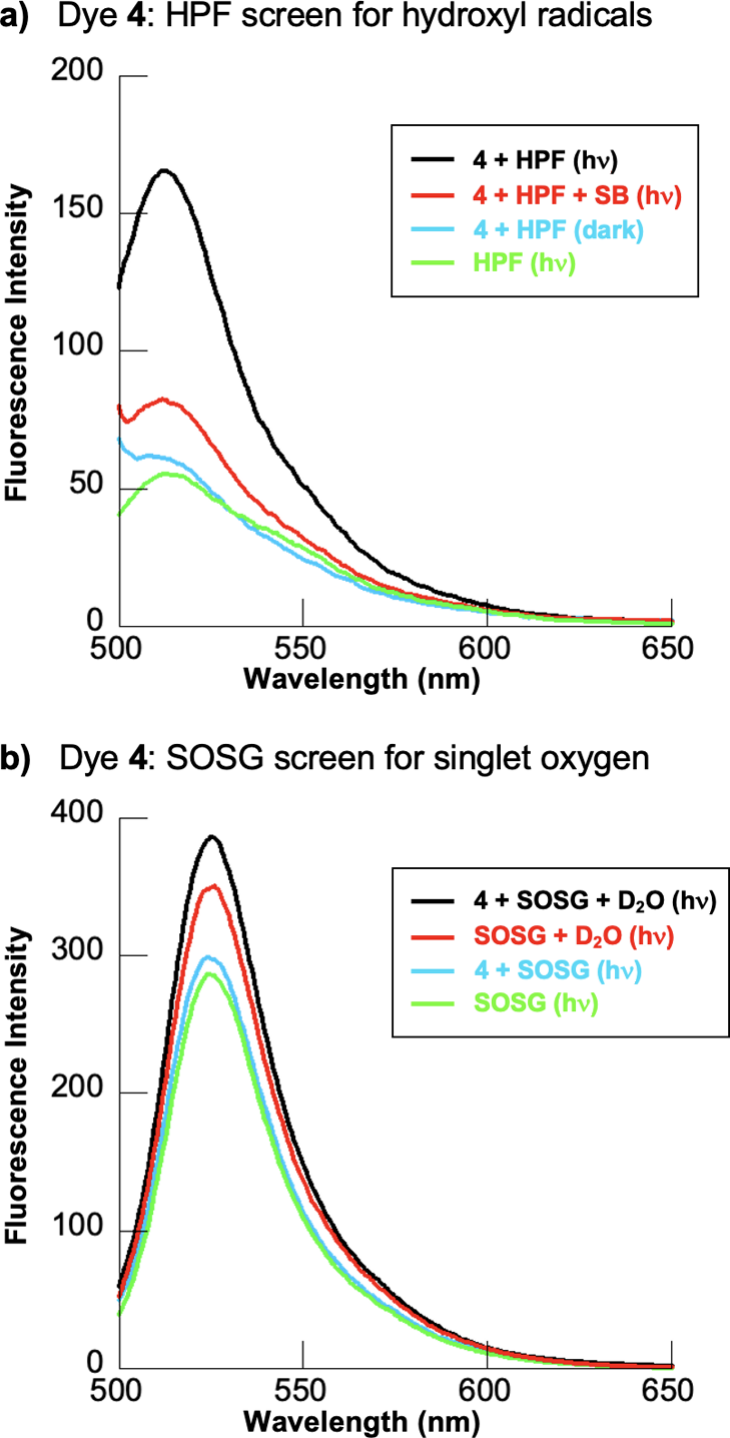
ROS probe fluorescence emission spectra. Reactions containing 10 mM sodium phosphate buffer pH 7.0 in the absence and presence of 10 μM dye **4** and either hydroxyphenyl fluorescein (HPF) or Singlet Oxygen Sensor Green (SOSG) were irradiated with an 850 nm, 0.9 W/cm^2^ LED laser for 30 min or kept in the dark (22 °C, no DNA). HPF: (a) concentrations of 3 μM HPF and 100 mM sodium benzoate were used to identify hydroxyl radicals. HPF emission spectra were recorded upon 490 nm excitation. SOSG: (b) concentrations of 0.75 μM SOSG and 90% D_2_O (v/v) were used to screen for singlet oxygen. SOSG emission spectra were recorded upon excitation at 480 nm.

In our next series of experiments, we respectively employed the fluorescent probes hydroxyphenyl fluorescein (HPF) and Singlet Oxygen Sensor Green (SOSG)^94,95^ as additional tests for dye **4**-photosensitized hydroxyl radical and singlet oxygen production. In so doing, reactions containing sodium phosphate buffer pH 7.0 in the absence and presence of 4-quinolinium dye **4**, HPF or SOSG, and sodium benzoate or D_2_O were either irradiated at 850 nm or kept in the dark (30 min, 22 °C). DNA was intentionally omitted from these solutions due to the propensity of nucleic acids to rapidly scavenge hydroxyl radicals and singlet oxygen. HPF and SOSG fluorescence emission spectra were then recorded (Figure 9). In the case of HPF, ^•^OH production was indicated. Hydroxyphenyl fluorescein is a widely used hydroxyl radical probe due to its high selectivity for this oxidizing agent, sensing ^•^OH, peroxynitrite anions (ONOO^–^), superoxide anions radicals (O_2_^•–^), hypochlorite anions, singlet oxygen (^1^O_2_), and hydrogen peroxide (H_2_O_2_) at respective ratios of 730:120:8:6:5:2.^96^ The maximum HPF emission intensity was achieved only when dye **4** was irradiated at 850 nm (Figure 9a, black line). Upon the addition of sodium benzoate to the solution, the fluorescence intensity was substantially decreased denoting the scavenging of dye-photosensitized hydroxyl radicals (Figure 9a, red line).

With respect to the singlet oxygen probe SOSG, the fluorescence emitted by irradiated reactions in H_2_O was roughly equivalent irrespective of whether dye **4** was included in the solution (Figure 9b, blue and green lines). When the neat water was replaced with 90% D_2_O (v/v), SOSG fluorescence was increased, pointing to the production of some singlet oxygen. However, the SOSG signal seen when dye **4** is present is only slightly more intense than the background (Figure 9b, black and red lines), suggesting that **4** does not generate substantial levels of ^1^O_2_. Taken together, the HPF and SOSG data in Figure 9 are consistent with the results of the chemical additive DNA photocleavage experiments shown in Table 1 and Figures S3 and S4. It is evident that irradiation of dye **4** at 850 nm primarily photosensitizes the production of DNA-damaging hydroxyl radicals.

### Photodynamic Effects of Dye **4** in *E. coli*

**Figure 10:**
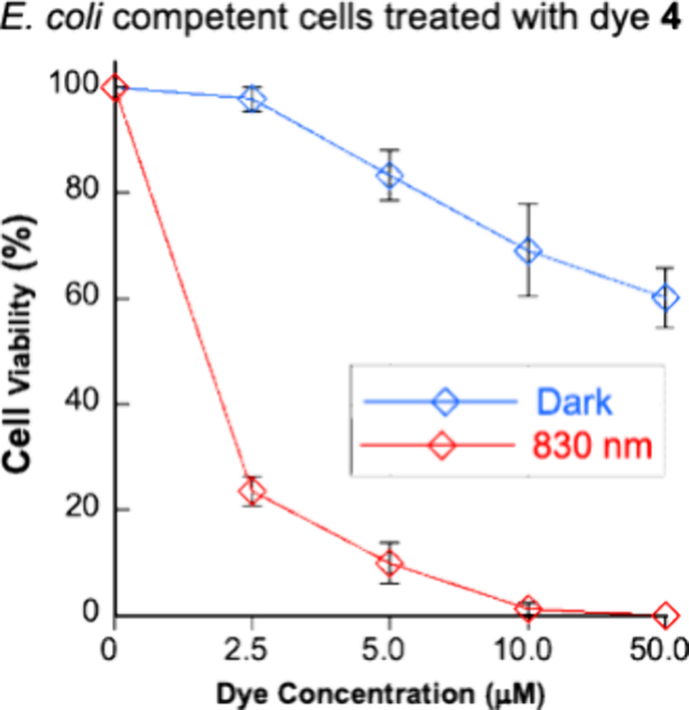
Final concentrations of 0–50 μM dye **4** were added to cultured *E. coli* strain DH5a cells in LB broth (*E. coli* optical density at 600 nm = 0.005; 40 μL total volume). After a 5 min equilibration period, the cells were either kept in the dark or irradiated with an 830 nm, 3.3 W/cm^2^ LED laser for 30 min. Reactions were then spread on LB plates and grown for 16 h at 37 °C in the dark. Colonies were counted with ImageJ software. All data points on the above plot represent the cell viability % averaged over 3–4 trials. Error bars indicate standard deviation.

To assess cultured *E. coli* DH5α cell growth, parallel solutions containing dye **4** concentrations ranging from 0 to 50 μM were equilibrated for 30 min, either in the dark or under 830 nm light exposure, plated, and then grown overnight.^97^ Cell viability percentages were then determined by counting surviving bacterial colonies. The results of this experiment revealed that dye **4** was substantially less toxic in the dark at all tested dye concentrations (Figure 10). Of note, 2.5 μM of dye **4** exhibited extremely low dark toxicity with a good photodynamic effect: a total of 24 ± 3% of cells survived under single-photon NIR illumination compared to 98 ± 2% of the dye-treated *E. coli* cells kept under dark conditions.

## Conclusions

The benefit of employing infrared light in photodynamic therapy rests in its ability to deeply penetrate biological tissues leading to superior activation of phototherapeutic agents. One trade-off associated with the use of deeply penetrating IR irradiation is imposed by the low triplet state energies associated with chromophores that absorb light in this wavelength range. To test the theoretical upper wavelength limit for an NIR agent to photosensitize the production of DNA-damaging ROS, here, we have synthesized and evaluated two symmetrical dicarbocyanine dyes in which redshifting benzylated 4-quinolinium rings are connected by a pentamethine bridge *meso*-substituted with either bromine (**4**) or hydrogen (**5**) (Scheme 1). While both dyes form direct DNA strand breaks when irradiated with a single-photon 850 nm LED laser (pH 7.0; Figure 2), the electron-withdrawing bromine atom of dye **4** increases DNA cleavage by substantially reducing dye autoxidation (Figures 1 and 2).

To establish relationships between dye **4** aggregation patterns, DNA binding modes, and the ability of the dye to photosensitize the formation of direct DNA strand breaks, UV–visible, circular dichroism, and fluorescence spectra were recorded (Figures 5–8). The data suggest that a putative bathochromic dye **4** monomer that engages in outside edge interactions with the DNA backbone is primarily responsible for the NIR photonuclease activity of the dye (Figures 2–4). In general, chromophores photosensitize ROS formation more efficiently when bound to DNA externally compared to snug intercalative and groove binding interactions that hinder access to ground state triplet oxygen (^3^O_2_).^28,74,98,99^ External binding may therefore contribute to the special ability of monomeric dye **4** to photosensitize the formation of highly reactive, DNA cleaving hydroxyl radicals in aqueous solutions (850 nm, pH 7.0; Table 1, Figure 9, and Figure S3 and S4).

In closing, we have shown that benzylated 4-quinolinium dicarbocyanine dye **4** forms DNA direct strand breaks when irradiated at NIR wavelengths as high as 905 nm, results that constitute the first published example of DNA photocleavage upon single-photon excitation above 830 nm (Figures 2–4). Dye **4** also functions as a good antibacterial agent, substantially reducing the viability of *E. coli* DH5α cells under 830 nm illumination (Figure 10). With future phototherapeutic applications in mind, we are now incorporating new design elements into third-generation cyanine-based photosensitizers. Our goals are to increase dye stability and to suppress the formation of hypsochromic dye aggregates in favor of ^3^O_2_-accessible bathochromic monomers that bind to DNA externally.

## Materials and Methods

### General

All reagents used in these experiments were of the highest available purity and were used without further purification. Sterilized deionized distilled water was employed to prepare all aqueous solutions. Ethylenediaminetetraacetic acid, UltraPure calf thymus DNA (10 mg/mL, average size ≤ 2000 bp), *E. coli* DH5α competent cells (used both in cytotoxicity studies and to clone the pUC19 plasmid), hydroxyphenyl fluorescein, and Singlet Oxygen Sensor Green were obtained from Thermo Fisher Scientific. Deuterium oxide (99.9%) and agarose were respectively purchased from Cambridge Isotope Laboratories and Bio-Rad. Bacto-tryptone, Bacto-agar, and Bacto-yeast extract were from Difco. All other reagents, including dimethyl sulfoxide (DMSO, ≥99.99%), lepidine (Sigma-Aldrich, 99%), sodium benzoate (SigmaUltra, minimum 99%), ethidium bromide, and pentamidine isethionate salt were provided by Sigma-Aldrich (St. Louis, MO, USA). The pUC19 plasmid was cloned according to standard laboratory protocols^100^ and was purified as per the manufacturer’s instructions using a QIAfilter Plasmid Mega Kit (Qiagen, Hilden, Germany; cat. no. 12263). Organic reactions were monitored using thin-layer chromatographic (TLC) plates (Sorbtech Silica XHL TLC plates w/ UV 254 and Universal Scientific Incorporated Alumina G Neutral). ^1^H NMR data were collected on a Bruker Avance (400 MHz) spectrometer using TopSpin 3.6.2 software. Melting points (MP, open Pyrex capillary) were measured on a MEL-TEMP instrument and were uncorrected. High-resolution mass spectra (HRMS) were obtained at the Georgia State University Mass Spectrometry Facility using electrospray ionization (ESI) positive mode in methanol with 0.1% formic acid with a Thermo Scientific Dionex Ultimate 3000 instrument. UV–visible absorption and fluorescence emission spectra were respectively acquired with PerkinElmer Lambda 35 and PerkinElmer LS55 spectrophotometers (PerkinElmer, Waltham, MA, USA). Circular dichroism spectra were recorded using a Jasco J-1500 spectropolarimeter (Easton, MD, USA).

### Synthesis of Benzyl Salt

#### 1-Benzyl-4-methylquinolin-1-ium Bromide (**3**)

A 1:1 ratio of commercially available lepidine and bromomethyl benzene was heated to reflux in acetonitrile at 90 °C for approximately 3 h. The reaction mixture was monitored *via* thin-layer chromatography using dichloromethane/methanol (25:1) as an eluent until the starting materials were consumed. Upon completion of the reaction, the solution was cooled down, and acetone was added to precipitate out the salt. The crude product was suction-filtered, washed with acetone and diethyl ether, and then dried under vacuum. Yield: 46%. MP: 194–196 °C. ^1^H NMR (400 MHz, DMSO-*d*_6_): δ 3.04 (s, 3 H), 6.37 (s, 2 H), 7.35 (m, 5 H), 8.01 (t, *J* = 7.7 Hz, 1 H), 8.19 (m, 2 H), 8.49 (d, *J* = 8.9 Hz, 1 H), 8.55 (d, *J* = 8.4 Hz, 1 H), 9.7 (d, *J* = 6 Hz, 1 H). ^13^C NMR (400 MHz, DMSO-*d*_6_): δ 20.36, 59.91, 120.20, 123.44, 127.61, 127.78, 129.14, 129.53, 129.65, 130.13, 134.62, 135.64, 137.30, 149.67, 160.08.

### Synthesis of Symmetrical Pentamethine Cyanine Dyes **4** and **5**

Two equiv of benzylated salt **3** and one equiv of a polymethine linker, either **1-Br** (prepared as previously described)^50^ or **1-H** (malonaldehyde bisphenylimine monohydrochloride, purchased from Sigma-Aldrich), were dissolved in acetic anhydride (5 mL) in the presence of a 5% catalytic amount of triethylamine (TEA). The synthesis of **1-Br** was performed by both conventional^50^ and microwave-assisted methods.^51^ In the conventional approach, the reaction was heated up to 70 °C for 1 h, whereas microwave irradiation was done at 100–110 °C for only 2 min. The formation of dyes **4** and **5** was monitored with a UV–visible spectrophotometer until absorption at ∼800–830 nm was obtained and a starting material peak at ∼450 nm completely disappeared. Upon cooling the reaction mixture to room temperature, ethyl acetate (5 mL) was added to precipitate the dyes. Each mixture was filtered under vacuum and purified *via* recrystallization in a minimal amount of methanol followed by dilution with diethyl ether causing precipitation. The crystals were isolated by vacuum filtration, dissolved in dichloromethane (5 mL), and precipitated with hexanes. The green solid products were collected and dried under vacuum.

#### 1-Benzyl-4-((1*E*,3*Z*)-5-((*Z*)-1-benzylquinolin-4(1*H*)-ylidene)-3-bromopenta-1,3-dien-1-yl)quinolin-1-ium Bromide (**4**)

Yields: 60 and 80% for the conventional and microwave approaches, respectively. MP: 193–195 °C. ^1^H NMR (400 MHz, DMSO-*d*_6_): δ 5.58 (s, 4 H), 6.98 (d, *J* = 12.7 Hz, 2 H), 7.3 (m, 6 H), 7.35 (t, *J* = 7.0 Hz, 4 H), 7.55 (d, *J* = 7.0 Hz, 2 H), 7.62 (t, *J* = 7.4 Hz, 2 H), 7.82 (m, 4 H), 8.31 (d, *J* = 12.7 Hz, 2 H), 8.38 (d, *J* = 8.5 Hz, 2 H), 8.54 (d, *J* = 7.4 Hz, 2 H). ^13^C NMR (100 MHz, DMSO-*d*_6_): δ 57.18, 109.53, 109.98, 118.56, 125.25, 125.42, 127.11, 127.20, 128.57, 129.42, 131.66, 133.50, 136.06, 138.62, 143.09, 144.03, 149.23. ESI-MS (positive mode) calculated for C_37_H_30_BrN_2_: *m*/*z* 581.1587, found: *m*/*z* 581.1585 (Supporting Information, Figures S5–S7).

#### 1-Benzyl-4-((1*E*,3*E*)-5-((*Z*)-1-benzylquinolin-4(1*H*)-ylidene)penta-1,3-dien-1-yl)quinolin-1-ium Bromide (**5**)

Yield: 65%. MP: 184–186 °C. ^1^H NMR (400 MHz, DMSO-*d*_6_): δ 5.69 (s, 4 H), 6.72 (t, *J* = 12.4 Hz, 1 H), 7.05 (d, *J* = 12.3 Hz, 2 H), 7.30 (t, *J* = 12.4 Hz, 6 H), 7.37 (t, *J* = 12.4 Hz, 4 H), 7.44 (d, *J* = 7.3 Hz, 2 H), 7.5 (t, *J* = 8.6 Hz, 2 H), 7.72 (m, 4 H), 8.04 (t, *J* = 12.7 Hz, 2 H), 8.33 (d, *J* = 7.3 Hz, 2 H), 8.43 (d, *J* = 8.4 Hz, 2 H). ^13^C NMR (100 MHz, DMSO-*d*_6_): δ 56.69, 108.84, 111.86, 118.13, 125.18, 125.60, 126.13, 126.43, 127.08, 128.44, 129.39, 132.98, 136.38, 138.69, 141.94, 146.91, 147.77. ESI-MS (positive mode) calculated for C_37_H_31_N_2_: *m*/*z* 503.2482, found: *m*/*z* 503.2479 (Supporting Information, Figures S8–S10).

### UV–Visible Spectroscopy

In the time course experiments in Figure 1, absorption spectra were recorded at intervals of 0 up to 30 min. Cuvettes contained either 10 μM dye **4** in neat DMSO or in 10 mM sodium phosphate pH 7.0 in the absence and presence of 150 μM bp of CT DNA (500 μL final volume, 22 °C). The remaining spectra were acquired in a similar fashion with the following procedural modifications. In Figure S1, spectra were recorded in DMSO for dye **4** and **5** concentrations of 1, 2, 3, 4, 5, and 10 μM. This was done to test for dye aggregation in DMSO and to predict the DMSO molar extinction coefficient at the λ_max_ of each dye by linear regression analysis. In the DNA titration experiment in Figure 5, small volumes of an aqueous solution of 13,088 μM bp of CT DNA were consecutively added to samples containing 20 μM dye **4** in 10 mM sodium phosphate pH 7.0 with an initial volume of 500 μL and a final volume of 563 μL. Concentrations of CT DNA present in the sample ranged from 34 μM up to 1328 μM bp. All titration absorption spectra were corrected for sample dilution. In the competitive DNA binding studies shown in Figure 8, UV–visible spectra were recorded for 500 μL solutions containing dye **4** and/or 50 μM of pentamidine in the absence and presence of 150 μM bp of CT DNA (22 °C).

### DNA Photocleavage

Reactions in Figure 2 contained 20 μM dye **4** or **5** with 38 μM bp of pUC19 plasmid DNA and 10 mM of sodium phosphate at pH 7.0 (40 μL total volume). The temperatures were controlled by transferring the Eppendorf tubes containing the reactions to a metal block that was placed in a 10 °C ice bath. The samples in the block were kept in the dark or were irradiated either with an 830 nm (3.3 W/cm^2^; Lilly Electronics), 850 nm (0.9 W/cm^2^; Lilly Electronics), or 905 nm, (0.5 W/cm^2^; CivilLaser) single-photon LED laser (30 min). Surface temperatures of individual reactions were recorded with a PREXISO PIX-500 noncontact infrared thermometer before and after the 30 min irradiation time or 30 min dark interval. Then, a total of 2 μL of the loading buffer containing 15.0% (w/v) ficoll and 0.025% (w/v) bromophenol blue was added to each sample and 20 μL of the resulting solutions were loaded into the wells of a 1.3% agarose gel containing a final concentration of 0.5 μg/mL of the DNA visualizing fluorophore ethidium bromide (EtBr). The reactions were then electrophoresed at 100 V for 60 min in a Bio-Rad Laboratories gel box containing a 1× tris-acetate-EDTA (TAE) running buffer and 0.5 μg/mL ethidium bromide. The gels were visualized at 302 nm with a VWR Scientific LM-20E transilluminator, photographed with a UVP PhotoDoc-It imaging system, and quantitated using ImageJ software (National Institutes of Health). In the case of supercoiled DNA, integrated numerical values were multiplied by a factor of 1.22 to correct for the decreased affinity of EtBr for supercoiled vs nicked and linear plasmid forms. The 1.22 correction factor, which was derived by the Dervan lab^101^ using the method of Haidle et al.*,*^102^ is constant in value irrespective of changes in the concentrations of any DNA ligands employed.^99,101−104^ DNA photocleavage percentages were calculated as follows: percent photocleavage = [(linear + nicked DNA)/(linear + nicked + supercoiled DNA)] × 100.

Subsequent photocleavage reactions were run as just described with the following modifications. In the case of the titration experiment shown in Figure 3, concentrations of dye **4** were increased from 0 to 45 μM. The Eppendorf tubes containing the reactions were transferred to a metal block that was placed either in the 10 °C ice bath or in a 37 °C heating unit and then kept in the dark or irradiated for 30 min. In the Figure 4 time course experiments, reactions containing 20 μM dye **4** were transferred to the metal block in a 10 °C ice bath and were then irradiated for intervals of time ranging from 0 to 90 min.

### Circular Dichroism

Samples used in the CD experiments in Figure 6 consisted of 20 μM cyanine dye **4** in 10 mM sodium phosphate pH 7.0 in the presence and absence of 0, 31, 241, 730, or 1283 μM bp concentrations of CT DNA (final volume, 2000 μL). Spectra were acquired from 200 to 800 nm in 3 mL, 1.0 cm path-length quartz cuvettes (Starna). The following instrument settings were employed: scan speed, 100 nm/min; response and digital integration times, 4 s; bandwidth, 2 nm; sensitivity, 200 millidegrees. The temperature was kept constant at 22 °C. Final spectra were the average of over 12 acquisitions.

### Fluorescence Spectrophotometry

To read dye **4** for fluorescence, solutions containing 10 mM sodium phosphate at pH 7.0 and 10 μM dye **4** in the presence or absence of 31 or 1283 μM bp of CT DNA (2000 μL total volume) were transferred to quartz cuvettes (Starna). Upon excitation at λ_ex_ values of 600, 660, 684, 790, or 800 nm, fluorescence emission was recorded at 22 °C, starting at a wavelength equal to λ_ex_ + 10 nm up to 800 or 900 nm (Figure 7). The scan speed of the fluorescence spectrophotometer was 100 nm/min, gain was set at medium, and the excitation and emission slit widths were both 4.5 nm.

In ROS detection experiments, fluorescence emission was recorded as just described with the following modifications. Reactions contained 10 mM sodium phosphate buffer pH 7.0 in the absence and presence of 10 μM dye **4** and either 3 μM^94,95,105,106^ of the hydroxyl radical probe hydroxyphenyl fluorescein (HPF) or 0.75 μM^15^ of the singlet oxygen probe Singlet Oxygen Sensor Green (SOSG; final volume of 2000 μL). DNA, which is an ROS scavenger, was excluded in all cases. Parallel HPF and SOSG reactions respectively contained 100 mM of the hydroxyl radical scavenger sodium benzoate and 90% D_2_O (v/v) to extend the lifetime of singlet oxygen. The reactions were then irradiated with an 850 nm, 0.9 W/cm^2^ LED laser for 30 min or kept in the dark (22 °C). Emission spectra were recorded from 500 to 650 nm upon 490 nm excitation of reactions containing HPF and 480 nm excitation of reactions containing SOSG (Figure 9).

### Reagent-Induced Changes in DNA Photocleavage

Solutions containing 10 mM sodium phosphate buffer pH 7.0 and 38 μM bp of pUC19 plasmid DNA were prepared in the absence and presence of 30 μM dye **4** and one of the following chemical additives: 50 μM pentamidine, 79% D_2_O (v/v), 100 mM sodium benzoate, and 100 mM EDTA in a total volume of 40 μL. The reactions were then irradiated for 30 min with an 850 nm, 0.9 W/cm^2^ LED laser or kept in the dark (Figures S3 and S4). All reaction temperatures were kept constant at 22 °C. After the irradiation, the cleaved DNA products were resolved on 1.3% nondenaturing agarose gels, visualized, and quantitated as just described. The percent inhibition of DNA photocleavage by each chemical additive was calculated using the formula percent inhibition = [((% linear + % nicked DNA with an additive) – (% linear + % nicked DNA without an additive))/(% linear + % nicked DNA without an additive)] × 100 (Table 1).

### Photocytotoxicity Assay

All solutions, media, and plasticware were sterilized in an autoclave before use. Initial 5 mL cultures of *E. coli* DH5α were grown for 8 h in Luria–Bertani (LB) broth under vigorous shaking at 37 °C. Enough of the starter culture (∼150 μL) was then added to the broth to give 450 μL of a bacterial solution with a confirmed optical density at 600 nm (OD_600_) of 0.5. This initial solution was then diluted 10-fold with LB broth to yield a final bacterial solution with an OD_600_ of 0.05. To determine phototoxicity, a total of 4 μL of this solution was added to an Eppendorf tube containing 30 μL of ddH_2_O, 4 μL of 100 mM sodium phosphate pH 7.0, and 2 μL of a dye/DMSO stock solution to afford a series of 40 μL reactions each containing 10 mM sodium phosphate buffer pH 7.0, a bacterial count equivalent to an OD_600_ of 0.005, and final dye **4** concentrations of 0, 2.5, 5, 10, or 50 μM. After a 5 min equilibration period, parallel reactions were either irradiated at 22 °C for 30 min by placing an 830 nm, 3.3 W/cm^2^ LED laser at the neck of each Eppendorf tube or were kept in the dark. Each of the 40 μL solutions was evenly spread on a separate LB agar plate with the aid of a spiral plater, incubated in the dark at 37 °C for 16 h, and photographed. The colonies on each plate were then counted using ImageJ software (National Institutes of Health). Cell survival percents corresponding to each plate were calculated using the equation (number of colonies on the plate with a dye/number of colonies on the control plate without a dye) × 100. Data were averaged over 3–4 trials with errors reported as standard deviation.

## Supporting Information Available

Dyes **4** and **5**: absorption in DMSO vs dye concentration, linear regression analyses, and absorption ratios; dye **4**-sensitized DNA photocleavage at 850 nm in the presence of pentamidine, D_2_O, Na^+^ benzoate, and EDTA; dye **4** and dye **5**^13^C and ^1^H NMR spectra and ESI mass spectra (PDF)

## Abbreviations

bp: base pair
CD: circular dichroism
CT DNA: calf thymus DNA
DMSO: dimethyl sulfoxide
EDTA: ethylenediaminetetraacetic acid
EtBr: ethidium bromide
ICD: induced circular dichroism
HPF: hydroxyphenyl fluorescein
LB: Luria–Bertani
LED: light-emitting diode
OD_600_: optical density at 600 nm
mtDNA: mitochondrial DNA
NIR: near-infrared
OY: oxazole yellow
PDT: photodynamic therapy
PS: photosensitizing agent
ROS: reactive oxygen species
S_1_: first excited singlet state
SOSG: Singlet Oxygen Sensor Green
TAE: tris-acetate-EDTA
TO: thiazole orange
