# Supplementary material for: Single-Photon DNA Photocleavage up to 905 nm by a Benzylated 4-Quinolinium Carbocyanine Dye
Source: ACS Omega. 2025 Feb 11;10(7):6544–58. doi: 10.1021/acsomega.4c07083 (PMC11865986; doi:10.1021/acsomega.4c07083)
Supplement: Supplementary file 1 — ao4c07083_si_001.pdf [file ao4c07083_si_001.pdf]

Supporting information

Single-Photon DNA Photocleavage up to 905 nm by a Benzylated 4-Quinolinium Carbocyanine Dye

Effibe O. Ahoulou,[1] Aikohi Ugboya,[1] Victor Ogbonna,[1] Kanchan Basnet,[1] Maged Henary,[1,2]* and Kathryn B. Grant[1]*

[1]Department of Chemistry, Georgia State University, [2]Center for Diagnostics and Therapeutics, Atlanta, GA 30303, United States

Contents:

Fig. S1. UV-visible spectra of 1 μM to 10 μM of dye in DMSO; linear regression analysis… S2

Fig. S2. Dye absorption in DMSO: λ_{max}/ absorption at hypsochromic shoulder……..…….. S3

Fig. S3. DNA photocleavage at 850 nm in the presence of pentamidine……......................... S4

Fig. S4. DNA photocleavage at 850 nm in the presence of D_2O, Na^+ benzoate, EDTA......... S4

Fig. S5. ^{1}H NMR spectrum of dye **4** ……………………………………………..…….. S5

Fig. S6. ^{13}C NMR spectrum of dye **4** ……………………………………………..….. S6

Fig. S7. High Resolution ESI mass spectrum of dye **4** …………………………………….. S7

Fig. S8. ^{1}H NMR spectrum of dye **5** ……………………………………………..…….. S8

Fig. S9. ^{13}C NMR spectrum of dye **5** ……………………………………………..……. S9

Fig. S10. High Resolution ESI mass spectrum of dye **5** …………………………………….. S10

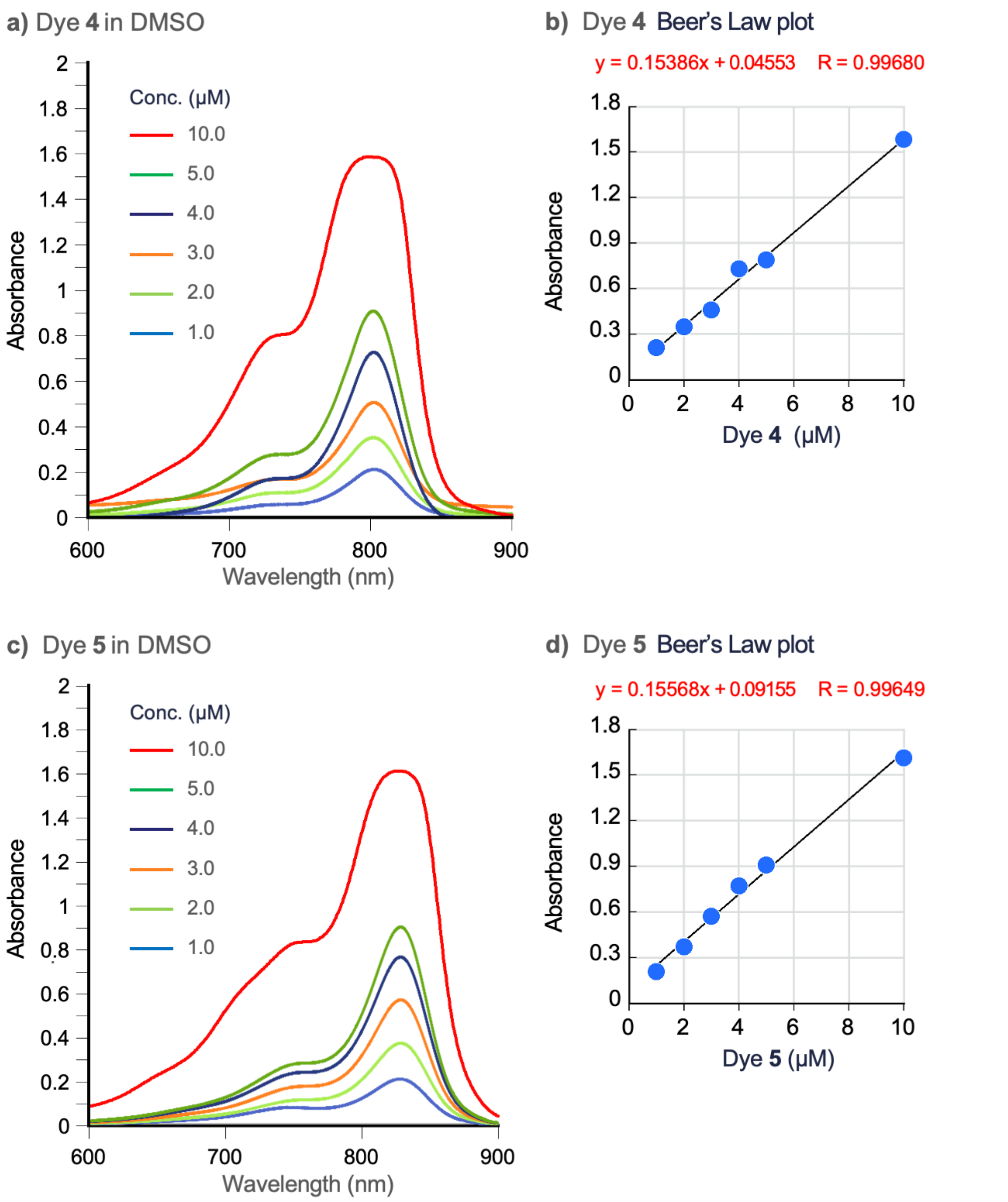

Figure S1. UV-visible absorption spectra in DMSO and corresponding λ_{max} absorbance plotted as a function increasing dye concentration for: **a, b)** dye **4** and **c, d)** dye **5**. Linear regression analyses (right) were used to fit the λ_{max} absorbance values to the best straight-line $y = mx + b$, where the slope m represents dye molar extinction coefficient in DMSO. Dye **4** absorbance values were adjusted to correct for the spectral baseline drift (*e.g.*, at 3 μM of dye). The 10 μM absorption spectra (red lines) are the 0 min spectra presented in Figures 1a and 1b of the main manuscript.

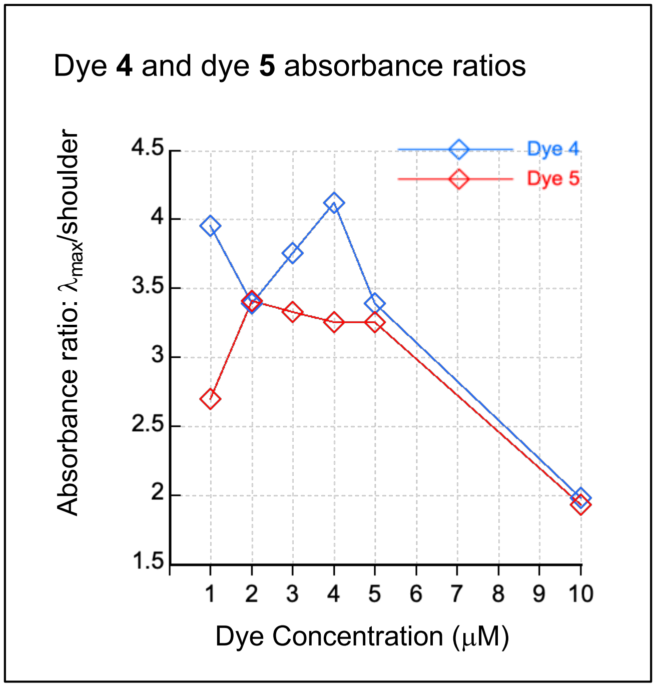

Figure S2. Absorbance ratios in DMSO: (absorption at the bathochromic λ_{max})/(maximum absorption at the hypsochromic shoulder for the dye **4** (blue line) and dye **5** (red line) absorption bands shown in Figure S1. In the case of dye **4**, absorbance values were adjusted to correct for the baseline drift of the spectra (Figure S1a).

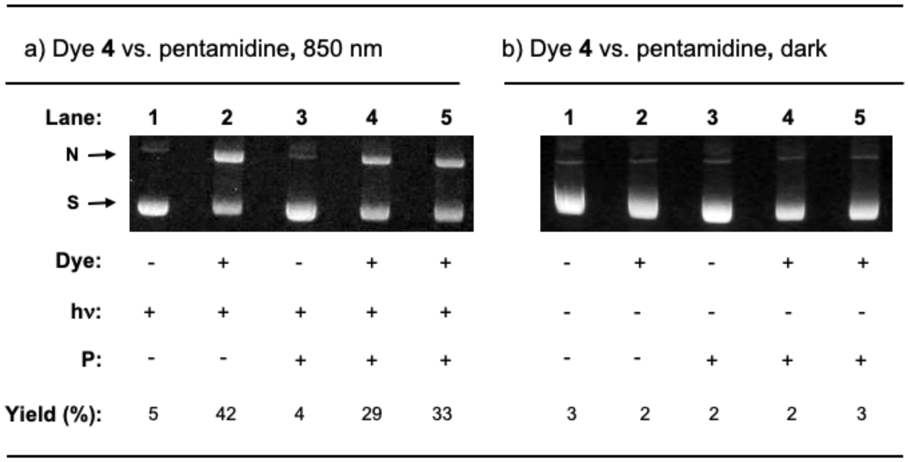

Figure S3. Representative ethidium bromide-stained agarose gel showing photocleavage of 38 μM bp pUC19 plasmid DNA in 10 mM sodium phosphate buffer pH 7.0. The DNA solutions were prepared with 30 μM of dye **4** in the presence and absence of 50 μM of pentamidine. For 30 min, individual reactions were either: **a)** irradiated with an 850 nm, 0.9 W/cm^2 LED laser or **b)** kept in the dark (10 °C). For each gel, the pentamidine was added either before (lane 4) or after (lane 5) dye **4**. Corresponding % photocleavage inhibition yields caused by pentamidine were averaged over 2-3 trials with errors reported as standard deviation (Table 1). Abbreviations: N, nicked; P, pentamidine; S, supercoiled.

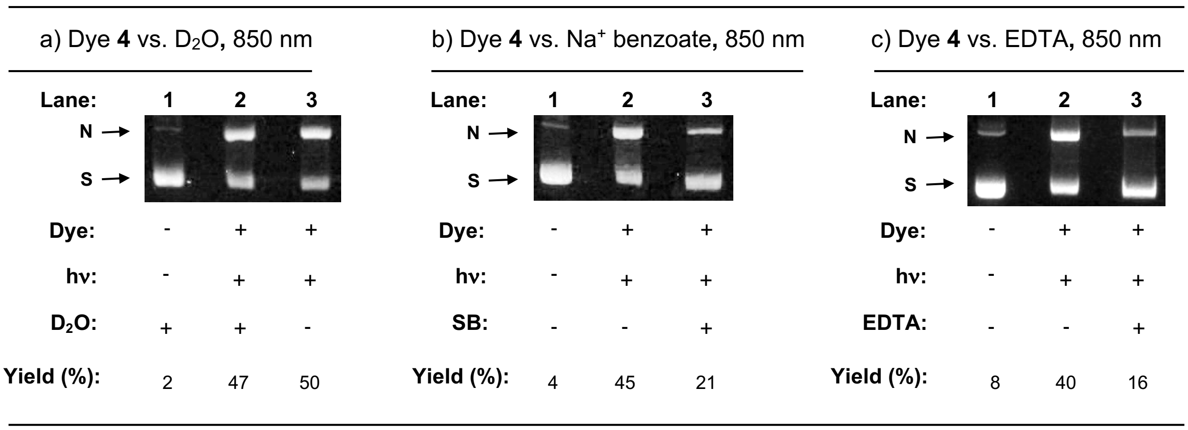

Figure S4. Representative ethidium bromide-stained agarose gels showing pUC19 plasmid DNA photocleavage. Reactions contained 10 mM sodium phosphate buffer pH 7.0 and 38 μM bp DNA in the absence and presence of 30 μM of dye **4** and a chemical agent: **a)** 79% *(v/v)* of deuterium oxide (D_2O); **b)** 100 mM of sodium benzoate (SB); **c)** 100 mM of ethylenediaminetetraacetic acid. Individual reactions were irradiated for 30 min with an 850 nm, 0.9 W/cm^2 LED laser or kept in dark (22 °C). Corresponding % photocleavage inhibition yields caused by each added reagent were averaged over 3 trials with errors reported as standard deviation (Table 1). Abbreviations: EDTA, ethylenediaminetetraacetic acid; N, nicked; SB, sodium benzoate; S, supercoiled.

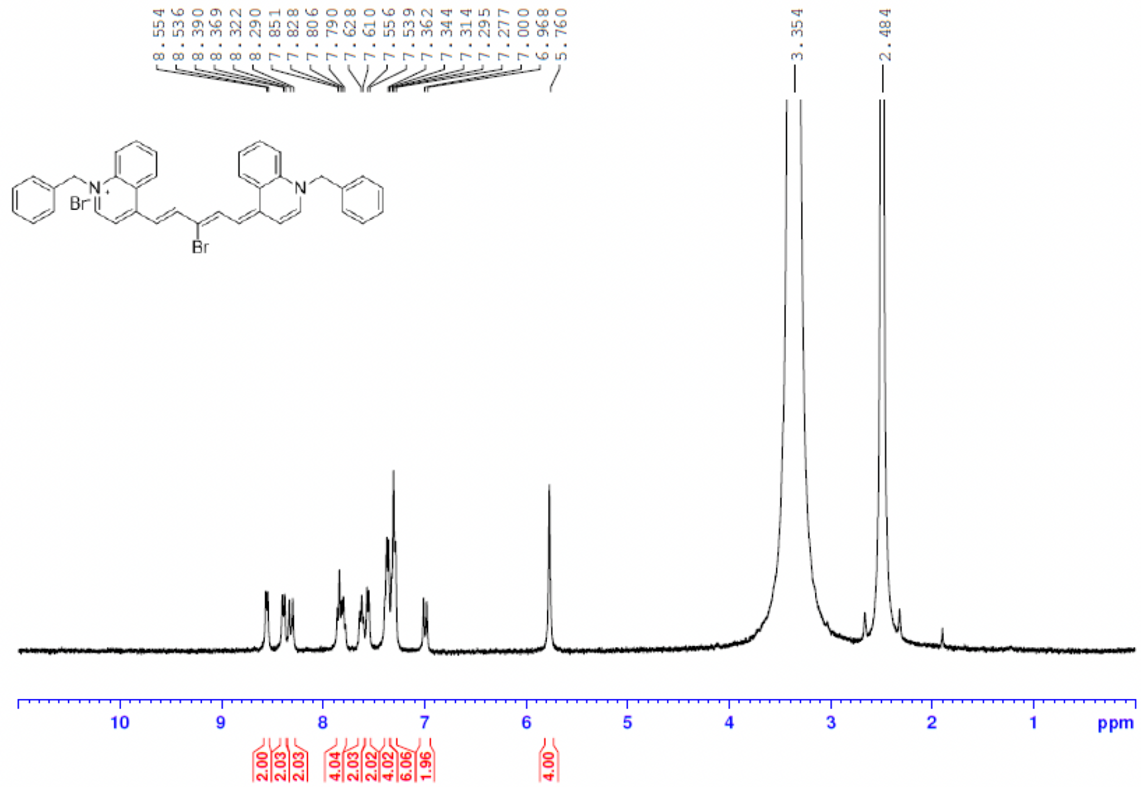

Figure S5. ¹H NMR spectrum of dye **4** (400 MHz, DMSO-d_6)**: δ** 5.58 (s, 4 H), 6.98 (d, *J* = 12.7 Hz, 2 H), 7.3 (m, 6 H), 7.35 (t, *J* = 7.0 Hz, 4 H), 7.55 (d, *J* = 7.0 Hz, 2 H), 7.62 (t, *J* = 7.4 Hz, 2 H), 7.82 (m, 4 H), 8.31 (d, *J* = 12.7 Hz, 2 H), 8.38 (d, *J* = 8.5 Hz, 2 H), 8.54 (d, *J* = 7.4 Hz, 2 H).

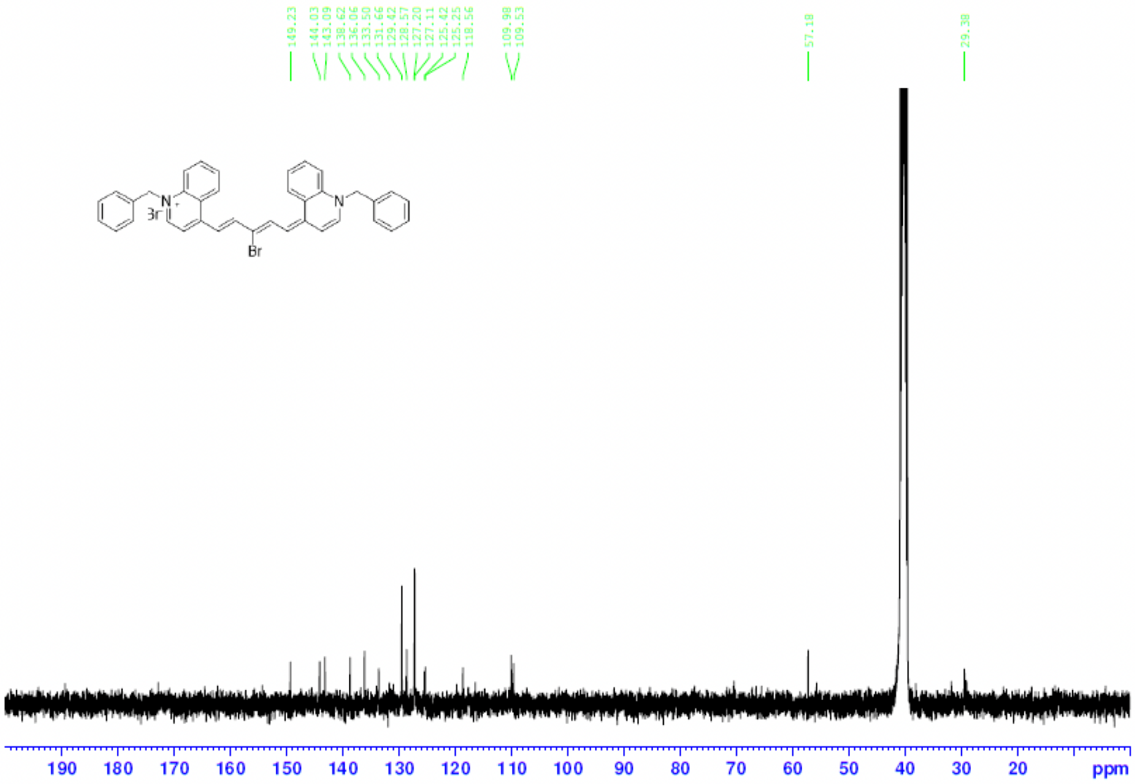

Figure S6. ^{13}C NMR spectrum of dye **4** (100 MHz, DMSO-d_6)**:** **δ** 57.18, 109.53, 109.98, 118.56, 125.25, 125.42, 127.11, 127.20, 128.57, 129.42, 131.66, 133.50, 136.06, 138.62, 143.09, 144.03, 149.23.

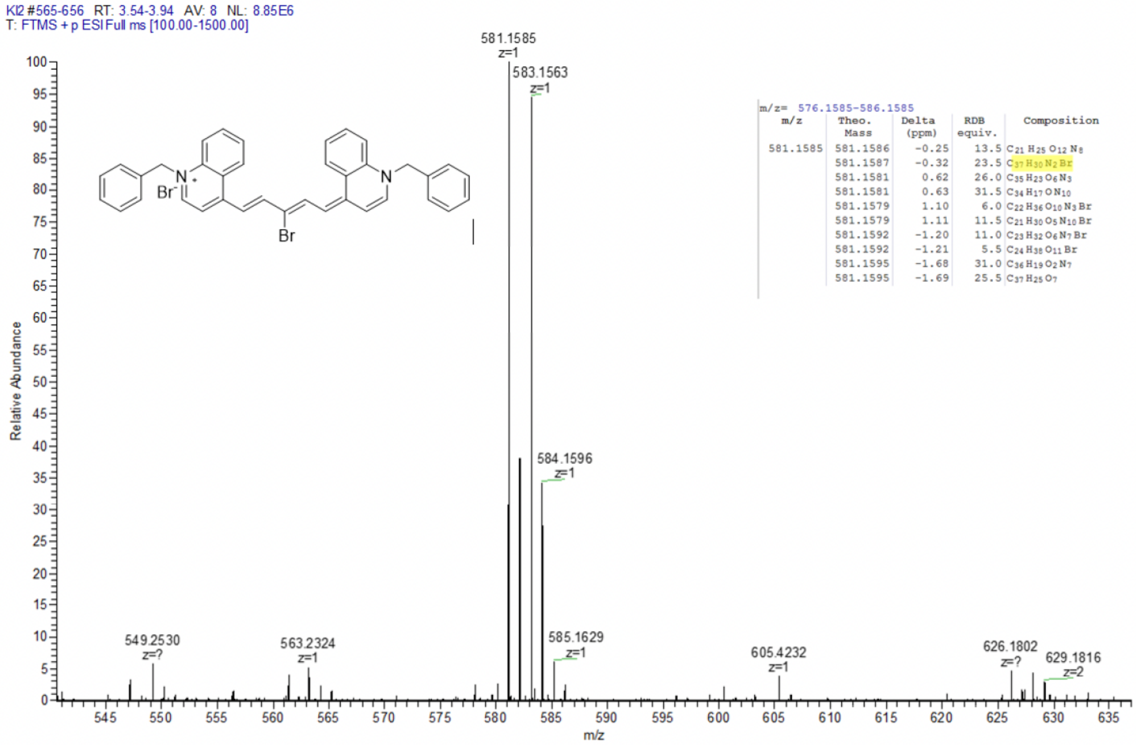

Figure S7. High Resolution ESI mass spectrum of dye **4** (positive mode) calculated for $C_{37}H_{30}BrN_2$: *m/z* 581.1587, found: *m/z* 581.1585.

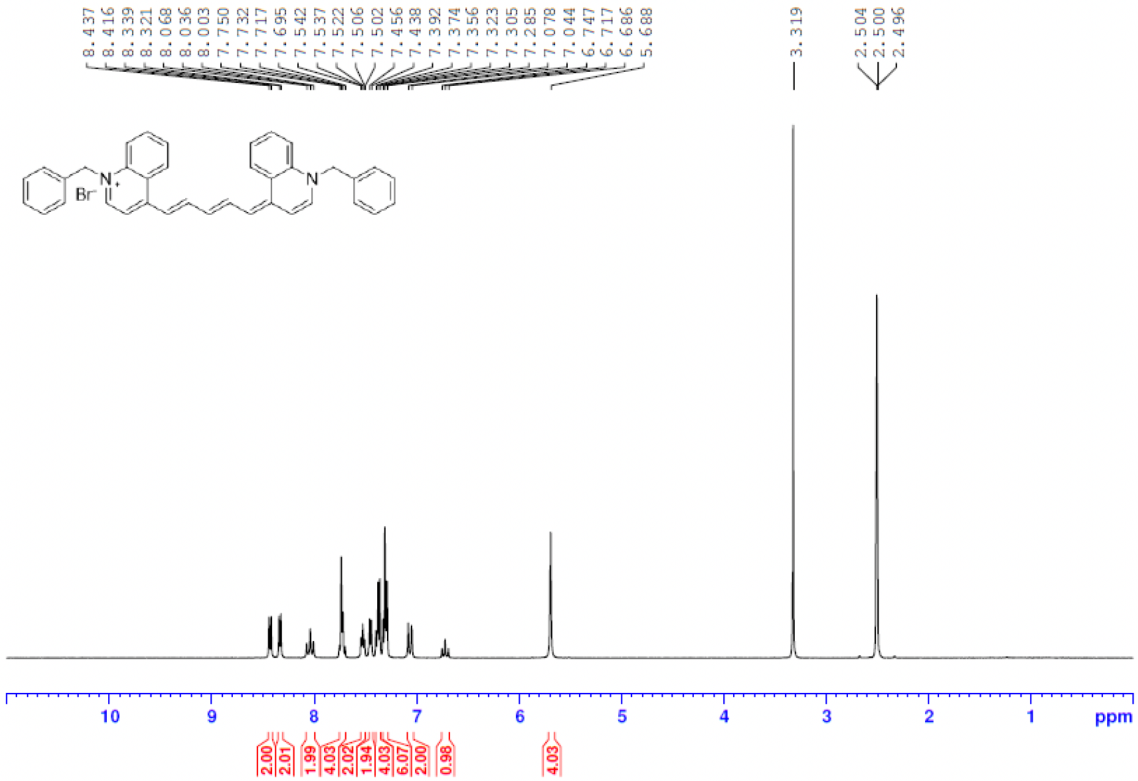

Figure S8. ^{1}H NMR spectrum of dye **5** (400 MHz, DMSO-d_6)**: δ** 5.69 (s, 4 H), 6.72 (t, J = 12.4 Hz, 1 H), 7.05 (d, J = 12.3 Hz, 2 H), 7.30 (t, J = 12.4 Hz, 6 H), 7.37 (t, J = 12.4 Hz, 4 H), 7.44 (d, J = 7.3 Hz, 2 H), 7.5 (t, J = 8.6 Hz, 2 H), 7.72 (m, 4 H), 8.04 (t, J = 12.7 Hz, 2 H), 8.33 (d, J = 7.3 Hz, 2 H), 8.43 (d, J = 8.4 Hz, 2 H).

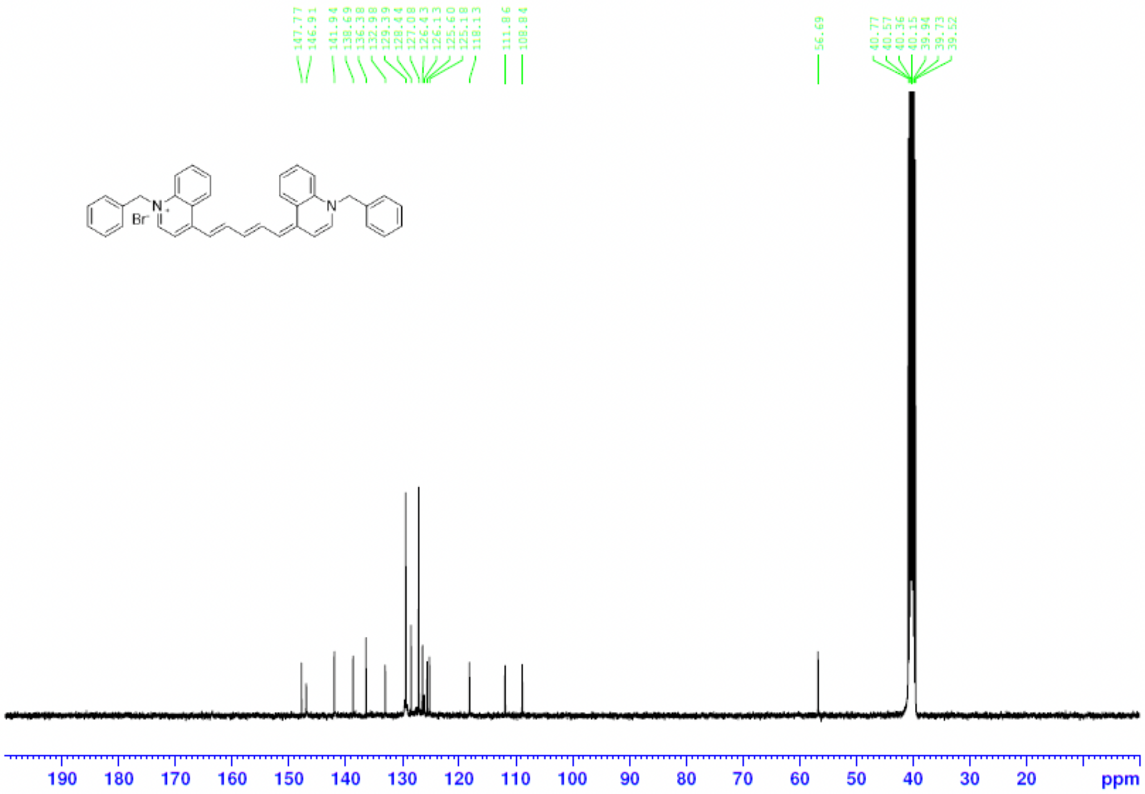

Figure S9. ^{13}C NMR spectrum of dye **5** (100 MHz, DMSO-d_6)**: δ** 56.69, 108.84, 111.86, 118.13, 125.18, 125.60, 126.13, 126.43, 127.08, 128.44, 129.39, 132.98, 136.38, 138.69, 141.94, 146.91, 147.77.

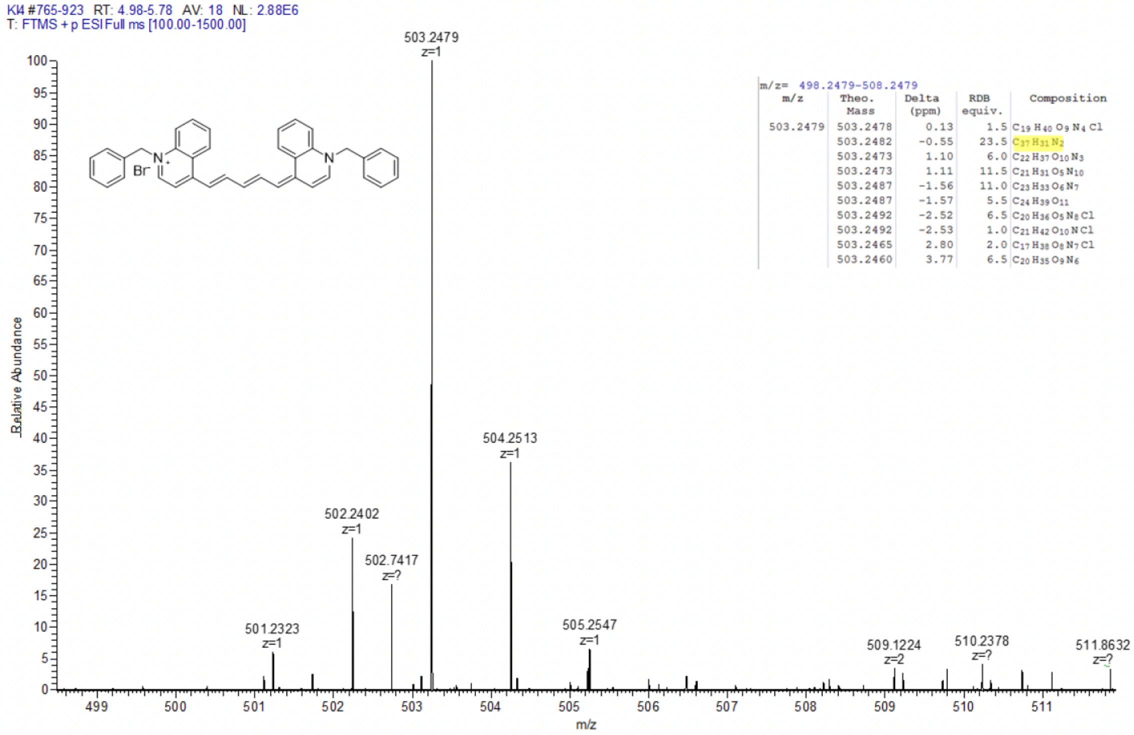

Figure S10. High Resolution ESI mass spectrum of dye **5** (positive mode) calculated for $C_{37}H_{31}N_2$: *m/z* 503.2482, found: *m/z* 503.2479.
